# How Health Care Professionals Use Social Media to Create Virtual Communities: An Integrative Review

**DOI:** 10.2196/jmir.5312

**Published:** 2016-06-16

**Authors:** Kaye Rolls, Margaret Hansen, Debra Jackson, Doug Elliott

**Affiliations:** ^1^ Agency for Clinical Innovation Intensive Care Coordination and Monitoring Unit NSW Health Department Chatswood Australia; ^2^ Faculty of Health University of Technology Sydney Sydney Australia; ^3^ Sydney Nursing School University of Sydney Sydney Australia; ^4^ School of Nursing and Health Professions University of San Francisco San Francisco, CA United States; ^5^ Oxford Brookes University Faculty of Health & Life Sciences Oxford United Kingdom; ^6^ Oxford University Hospitals Oxford Institute of Nursing & Allied Health Research NHS Foundation Trust Oxford United Kingdom; ^7^ University of New England School of Health Armidale Australia

**Keywords:** social media, literature review, physicians, nurses, midwives, social networking, pharmacist, social worker, allied health personnel

## Abstract

**Background:**

Prevailing health care structures and cultures restrict intraprofessional communication, inhibiting knowledge dissemination and impacting the translation of research into practice. Virtual communities may facilitate professional networking and knowledge sharing in and between health care disciplines.

**Objectives:**

This study aimed to review the literature on the use of social media by health care professionals in developing virtual communities that facilitate professional networking, knowledge sharing, and evidence-informed practice.

**Methods:**

An integrative literature review was conducted to identify research published between 1990 and 2015. Search strategies sourced electronic databases (PubMed, CINAHL), snowball references, and tables of contents of 3 journals. Papers that evaluated social media use by health care professionals (unless within an education framework) using any research design (except for research protocols or narrative reviews) were included. Standardized data extraction and quality assessment tools were used.

**Results:**

Overall, 72 studies were included: 44 qualitative (including 2 ethnographies, 26 qualitative descriptive, and 1 Q-sort) and 20 mixed-methods studies, and 8 literature reviews. The most common methods of data collection were Web-based observation (n=39), surveys (n=23), interviews (n=11), focus groups (n=2), and diaries (n=1). Study quality was mixed. Social media studied included Listservs (n=22), Twitter (n=18), general social media (n=17), discussion forums (n=7), Web 2.0 (n=3), virtual community of practice (n=3), wiki (n=1), and Facebook (n=1). A range of health care professionals were sampled in the studies, including physicians (n=24), nurses (n=15), allied health professionals (n=14), followed by health care professionals in general (n=8), a multidisciplinary clinical specialty area (n=9), and midwives (n=2). Of 36 virtual communities, 31 were monodiscipline for a discrete clinical specialty. Population uptake by the target group ranged from 1.6% to 29% (n=4). Evaluation using related theories of “planned behavior” and the “technology acceptance model” (n=3) suggests that social media use is mediated by an individual’s positive attitude toward and accessibility of the media, which is reinforced by credible peers. The most common reason to establish a virtual community was to create a forum where relevant specialty knowledge could be shared and professional issues discussed (n=17). Most members demonstrated low posting behaviors but more frequent reading or accessing behaviors. The most common Web-based activity was request for and supply of specialty-specific clinical information. This knowledge sharing is facilitated by a Web-based culture of collectivism, reciprocity, and a respectful noncompetitive environment. Findings suggest that health care professionals view virtual communities as valuable knowledge portals for sourcing clinically relevant and quality information that enables them to make more informed practice decisions.

**Conclusions:**

There is emerging evidence that health care professionals use social media to develop virtual communities to share domain knowledge. These virtual communities, however, currently reflect tribal behaviors of clinicians that may continue to limit knowledge sharing. Further research is required to evaluate the effects of social media on knowledge distribution in clinical practice and importantly whether patient outcomes are significantly improved.

## Introduction

Although modern health care organizations are purported to be knowledge intensive [1], current management structures and work practices do not always facilitate development of intellectual and structural capital [2] or innovation uptake, leading to challenges for translating research into practice (TRIP) [3]. Contemporary organizational [1] and learning theories [4] highlight learning and behavior as being socially constructed and therefore influenced by social networks [5]. However, despite implementation of clinical network structures aimed at improving patient care and facilitating knowledge sharing between health care professionals and across organizational boundaries, bureaucratic, hierarchical, and intraprofessional barriers persist [6].

Information technology and the Internet have revolutionized communication to such an extent that humans can now communicate with colleagues anywhere at any time using social media platforms. Within the health care literature, there are however polarized views regarding the benefits and negative aspects of professional social media use [7,8]. Given this evolving technological environment and related continuing professional debate, the purpose of this paper was to review the literature on the use of social media by health care professionals for facilitating professional networking, knowledge sharing, and evidence-informed practice. Theoretical frameworks used to embed the use of social media in enabling collegial networking, knowledge sharing, and supporting evidence-informed practice are explored in the following section for context, before the focused literature search and review.

### Background

Professional networking is a process of establishing a mutually beneficial relationship with other like-minded professionals [9]. For an organization, professionals networking between separate operational units promote knowledge flow and diffusion of innovations, potentially leading to improved professional performance [3]. Evolving views of learning including community of practice [4] and connectivism [10] highlight that professional development can be achieved through collective learning within social groups or networks. With the creation of Web-based communities, social media apps may facilitate this networking and professional development, enabling interactions between individuals regardless of time, space, or geography [11,12]. The interrelated concepts and frameworks are described in the following section as background for exploring this topic area: diffusion of innovations, learning theories, evidence-based practice, knowledge management, and work in health care practice and social media.

### Diffusion of Innovations

This theory describes how a novel idea, practice, or object is adopted by a particular social group or network [13]. In health, these innovations include new equipment, research findings, or practices. Rogers [13] demonstrated that although heterophilous communication (when individuals do not share common attributes such as values or socioeconomic status) increases access to novel ideas, for the vast majority of individuals, adoption of an innovation is dependent on homophilous communication (when individuals share common attributes). Five distinct types of individuals in a social group were identified. “Innovators” and “early adopters” are the first to adopt innovations with use mediated by a higher income and having greater access to novel information because of their broader, heterophilous social networks. The “early majority” are in turn influenced to adopt practices by observing use of and/or recommendation by early adopters. Finally, the “late majority” and “laggards” are the last to adopt because their communication channels are limited to those that share their views and experiences (homophilous) and are unlikely to be exposed to nonredundant knowledge or differing opinions [5,14].

Contemporary understanding of diffusion of innovations acknowledges that organizational or group factors also exert a powerful influence on individuals and the organization [15-17]. In particular, interconnectedness (connections between organizational members and units) and external orientation (organizational leaders with external networks) are both mediated by communication channels (networking internally or external to the organization) [13,16,17]. Individual innovators and early adopters with communication channels outside their everyday social and professional networks will learn more new information [18-20], although unless these individuals hold a central position within their local social network, it is unlikely this new knowledge will become embedded locally [14]. Credibility of intrapersonal channels (eg, peer to peer or opinion leader to professional) makes these channels more influential on adoption decisions [13,15,18,21,22]. Current social networks in health care organizations are generally homophilous with strong professional boundaries [23,24], which tend to control clinical practice [25].

### Learning Theories

Current views of learning also highlight the importance of interaction or networking between individuals for learning and professional development. As a social learning theory, community of practice (CoP) positions learning as a fundamentally social behavior where individuals learn through their interactions and participation in the world [4]. Within a CoP, members acknowledge a shared knowledge domain (craft knowledge), practice, and identity [4]. Professional development therefore occurs during everyday workplace interactions, where important “how to” knowledge can only be gained from other colleagues [26]. For health care professionals, CoP is particularly relevant as the theory provides a framework for understanding the professional development of individuals within the workplace through different forms of participation [4,27,28]. At present, however, the effectiveness of health care CoPs to facilitate professional development and improve clinical practice needs further investigation because projects to date have operationalized and measured the effectiveness of the CoP in different ways [29,30].

### Evidence-Based Practice

Recent literature on adoption of evidence-based practice [3] suggests that traditional health care structures do not create learning organizations that support: (1) development of intellectual capital [2]; (2) knowledge work [31]; or (3) assimilation of research findings into practice [32]. Furthermore, as knowledge does not flow freely between the silos of academia, clinical practice, publishing, and health care organizations, variations in the types and quality of care are common [33]. In health care, there have been mixed results where these channels (eg, opinion leaders) have been used to promote evidence-base practice [34,35] and peer-to-peer communication becomes more important as final adoption decisions are made [21]. In practice, however, clinicians continue to rely on personal knowledge (gained through education and experience) before seeking advice from close credible colleagues [36-39], despite the veracity of this advice not being critiqued or evaluated [36].

### Knowledge Management, Knowledge Work, and Health Care Practice

Currently, organizational productivity [40], improved health outcomes, and cost-effectiveness are linked to the presence of a definitive knowledge management strategy that supports activities of “knowledge workers” [41]. Contemporary knowledge management strategies focus on human and contextual elements of knowledge, such as how knowledge is created and diffused through an organization [42,43]. Interorganizational and intraorganizational networks are central to knowledge creation and diffusion, given that much knowledge is experiential, implicit, or tacit [44], particularly in health care organizations.

Knowledge work involves evaluating data from novel situations and applying specialized and expertise transfer, to discover or create knowledge in a given context [45]. Health care professionals (nurses, physicians, and allied health disciplines) are a subgroup of knowledge workers identified as “technologists,” where a personal knowledge store, initially based on formal academic education, evolves through experience and professional development [2]. Knowledge work can therefore be viewed as a form of evidence-based practice because it is the active thoughtful mode of work where clinicians decide how best to apply current knowledge, both personal and evidence, to individual patient care and other practice situations.

### Social Media

Computers, the Internet, and social media have revolutionized human communication [46]. Web 1.0, existing between 1980 and 2000, was a Web-based environment characterized by static webpages with centralized creation, control, and distribution of content [47] and user interactivity facilitated by early social media (discussion forums, bulletin boards, and Listservs) [48]. The range of social media platforms exploded with arrival of Web 2.0, enabling new technologies including social and professional networking sites (eg, Facebook and LinkedIn), thematic networks, microblogs, wikis, social photo and video sharing tools, collaborative filtering tools, and multiuser virtual environments [49-51].

Aided by diffusion of tablet technology, Internet access, and improved mobile connectivity, use of social media has increased exponentially over the past few years. Between 2015 and 2016, both Internet and social media users increased by 10% to 46% (3.419 billion) and 31% (2.307 billion), respectively; there are however significant regional and national differences [52]. With respect to Internet use, Iceland has the highest penetration (98%) followed by Bermuda (97%) and Norway, Denmark, Andorra, and United Arab Emirates next (96%). North Korea has the lowest population usage (0.03%) followed by a number of Central African countries with less than 5%. Active population use of an social media account is greatest in North America (59%), South America (50%), East Asia, and Western Europe (48% each) and lowest in Central Asia (6%) and South Asia (11%). Social media use is similar across Western nations (eg, 58% Australia, 59% United States, 59% United Kingdom) but less in China at 47%. Although Facebook continues to dominate the social sphere, with 1590 million active accounts, users appear to be gravitating toward apps for networking including WhatsApp (900 million), QQ (860 million), and Facebook messenger (800 million). Among other platforms, Tumblr, Instagram, and Twitter continue to experience growth, whereas Skype and LinkedIn are stable [52]. For this paper, we adopted the International Medical Informatics Association’s [51] classification, which identifies 13 types of social media platforms (see Textbox 1).

**Social media types [51].**

- Social networks
- Professional networks
- Thematic networks
- Microblogs
- Blogs
- Wikis
- Forums or Listserv
- Social photo and video sharing tools
- Collaborative filtering tools
- Multiuser virtual environments
- Social apps and games
- Integration of social media with health information technologies
- Other (eg, FriendFeed)

Importantly, not all social media apps have the functionality to promote development of a Web-based or virtual professional community. The success of interactive conversational technologies (including discussion forums, Listservs, wikis, blogs, microblogs, and social networking sites (SNS), is contingent on members joining and participating in ongoing interaction; these are therefore the main types of social media platforms capable of creating virtual communities. Although virtual communities have been examined by a number of researchers from different disciplines, at this time, there is no universally accepted definition [53]. For this paper, we define a Web-based (virtual) community as “… a group of people who share a strong common interest, form relationships and interact online” [53] (p. 3). A community’s existence depends on the structural capital produced from relationships established by member interaction and sharing of resources through the network [54]. Increasing numbers of organizations, professionals, and patients are now using social media to communicate and interact both internally and externally [55]. These real-life virtual communities or networks created by social media establish intrapersonal communication channels, overcoming barriers of time and geography, empowering users to communicate and interact (network) with a broad range of colleagues [11].

The purpose of this review was therefore to examine the research literature to identify how health care professionals use social media to develop virtual communities that facilitate professional networking, knowledge sharing, and evidence-informed practice. This review will add to the current literature by developing an understanding of how health care professionals use social media on a purely voluntary basis including integration of new media and behaviors such as conference tweeting.

## Methods

Within the context of learning theories, diffusion of innovation, and social media in health care, an integrative literature review [56] was conducted to evaluate whether health care professionals have been able to effectively leverage social media platforms to develop virtual professional communities that facilitate professional networking, knowledge sharing, and evidence-informed practice.

### Literature Search

Two major electronic health databases, CINAHL and PubMed, were searched for research papers published between January 1990 and December 2015. Keywords were used as they applied to the main concepts of social media, networking, and professional development including virtual communities, social media, computer-mediated communication, Listserv, discussion forum, networking, Twitter, and Facebook. Additional search strategies included a review of reference lists of the papers and handsearching the table of contents of key journals (see Multimedia Appendix 1 for detailed search).

Papers that fulfilled the following criteria were selected for review: involved HCP participation exclusively as a voluntary activity; English language; peer-reviewed; and all research designs that highlighted HCP interaction using social media to develop a virtual community as the core component. Social media included were Listservs, discussion forums, SNS, and microblogs. Papers were excluded if they: (1) described a project within an education context including undergraduate or postgraduate learning or organizational education or training; (2) study protocol; and (3) narrative review. The first author extracted data from studies using a standard data extraction tool [57].

### Study Methods Evaluation

After data extraction, the quality of each study was evaluated by 2 authors using standardized criteria. The CASP appraisal tool was used for qualitative studies (not including studies using content analysis) [58]. For studies using content analysis techniques [59-61], this included:

1. Data: appropriateness to research question, data corpus, sampling unit, unit of analysis, and sampling plan (described and justified).

2. Coding schema: appropriateness of approach, development, coders, training, theoretical underpinning of categories, and reliability of coding schema.

3. Analysis: appropriateness of approach.

Quality criteria for surveys included: (1) research question and design; (2) sampling framework and participant understanding; (3) instrument metrics; (4) response rate; (5) coding and analysis; and (6) result presentation [16]. The Scottish Intercollegiate Guidelines Network [62] appraisal tool was used for literature reviews. Studies were categorized as strong (most elements described with satisfactory quality), moderate, fair, and limited (poor reporting or description of research method).

### Data Analysis

After data extraction and evaluation of study quality, summary tables were constructed to reduce data into manageable frameworks [56] and facilitate identification of patterns. These tables included data pertaining to:

1. Research overview including context, social media type, research design, sample and/or data corpus, data analysis and quality.

2. Web-based behavior including manifest and latent characteristics of emails or tweets and posting habits of members.

3. Reasons for belonging to a virtual community including meaning or value of community to members and motivators and barriers to Web-based participation.

4. Descriptions of Web-based communities including context, membership, and reasons or objectives for establishing Web-based community.

5. Research examining general social media use.

Only a limited amount of quantitative data could be aggregated for comparison across studies because of different data collection methods and outcomes. Qualitative data were synthesized to identify consistent patterns and themes.

### Findings

**Figure 1:**
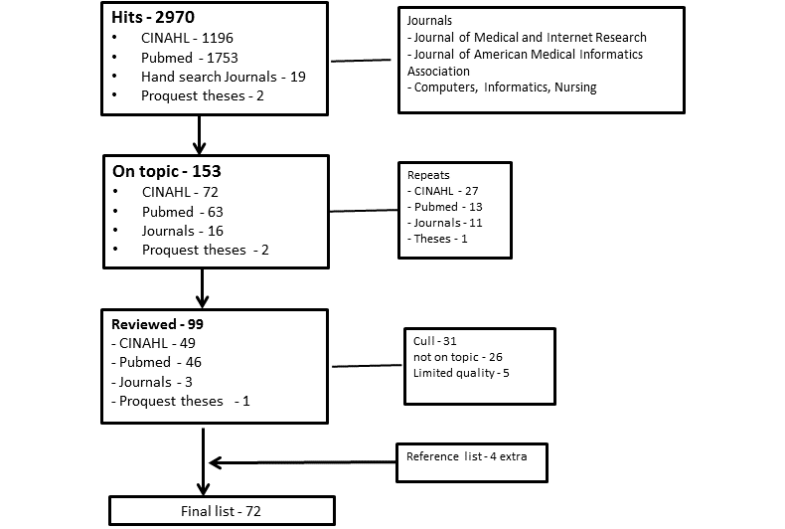
Literature search using PRISMA guidelines [63].

Overall, 72 studies were included in the final review (see Figure 1 [63] and Multimedia Appendix 2). An overview of studies including context, design, instruments and data collection, sample and data corpus, data analysis, and study quality is listed in Multimedia Appendix 2). Findings are presented in the following sections:

1. Overview of research methods and critique of study quality

2. Social media use by health care professionals.

3. Web-based posting behaviors including the manifest and latent content of communication (emails, posts, or tweets) and posting habits.

4. Mediating factors of Web-based posting.

### Overview of Research Methods and Critique of Study Quality

Of the 72 studies selected, there were 44 qualitative, 20 mixed methods, and 8 literature reviews. The most common methods of data collection were Web-based observation (n=30 studies), surveys (n=18), interviews (n=12), and focus groups (n=2).

Qualitative methods included: (1) qualitative (n=14; survey 11; discourse analysis 1; and interviews 2); (2) qualitative descriptive (n=26; content analysis 19, descriptive 5; thematic 1; social network analysis 1); (3) ethnography (n=2), Q-sort (n=1), and social network analysis (n=1). Q-sort is a multilevel study method where qualitative (subjective) responses are refined to develop a quantitative understanding or hierarchy of the phenomenon of interest [128]. Of the 20 mixed-method studies, combinations of methods included: content analysis and interviews (n=5); content analysis and survey (n=3); content analysis, survey, and social network analysis (n=1); Web-based observation and thematic analysis (n=1); Web-based observation and social network analysis (n=2); survey and diaries (n=1); survey and interviews (n=2); survey, interviews and observation (n=1); and survey and Web-based observation (n=2). Overall, the quality of these qualitative studies was satisfactory, with most fulfilling the CASP criteria [58] (see Multimedia Appendix 3). The quality of studies using content analysis (see Multimedia Appendix 4), survey methods (see Multimedia Appendix 5), or literature review (Multimedia Appendix 6) was mixed.

### Content Analysis

Content analysis was commonly used in studies to reveal the content and meaning of textual data, which remains embedded in its origin or context [59]. In relation to Web-based communication, this approach can reveal the acquisition of new knowledge and skills and the social construction of knowledge [64]. A total of 30 studies used Web-based observation to collect emails, discussion threads, or tweets and applied either inductive (n=10) or deductive (n=20) content analysis techniques to identify: manifest content (topic, type of post, type of knowledge, frequency, discussion thread length, and/or participation rate) and latent content (accuracy of information, presence of knowledge work, or sophistication of discussion). Listservs or mailing lists (n=15) were the most common social media type examined followed Twitter (10), discussion forums (n=3), Web-based journal clubs (n=1), and Facebook (n=1).

The quality of studies was evaluated as high quality (n=12) or moderate quality (n=8), with the remaining 10 only fulfilling a limited number of required criteria (See Multimedia Appendix 4). Common study limitations affecting the validity of results included failure to report or justify the following elements: (1) data corpus and/or sampling unit [65-69]; (2) unit of analysis [65-67,69-74]; (3) coding schema development and categories with a limited theoretical basis for categories [66,68,69,73,75-77]; and (4) evaluate inter-rater reliability [66,68,69,71,73,78-80]. Only 2 studies kept the unit of analysis (that is post or email) within its contextual unit (ie, discussion thread) [77,81]. Sampling methods to gather the data corpus for analysis varied considerably. Most reports describe using a census sample [31,65,66,70,71,76,77,79,82-86] with stratified [27,80,87-91] or convenience samples [67-69,75,81,92,93] were used less often. A random sample was used only once [74]; however, this was not adequately described.

### Surveys

A survey design was used by 23 studies to examine member experiences with or intentions to use social media; only 2 demonstrated strong quality, 9 were moderate quality, and 12 were fair quality (see Multimedia Appendix 5). Methodological limitations impacting on the validity and generalizability of these findings included: (1) limited information regarding survey tool development [66,94-104] and (2) sampling bias including recruitment methods, low response rate, and/or failure to identify whether respondents were representative of community membership or study population [66,83,96,99,101-109].

### Literature Reviews

Eight literature reviews were identified (4 systematic; 2 scoping; and 2 with no specific descriptor) with variable quality demonstrated (see Multimedia Appendix 6) [62]. The main deficits were: limited description of method [110,111]; a search strategy that was limited by years and/or databases [110-112]; and failure to evaluate the quality of studies covered [111-115]. Although each review had different questions, there were overlapping content areas: (1) social media adoption by clinicians [110], pharmacists [112,113], and radiographers [111]; (2) social media use for communication between patients, patient–clinician, or clinicians [116]; (3) type of social media use by clinicians [115]; (4) virtual communities for general practitioner professional development [12]; and (5) Twitter journal clubs [114]. Two studies [115,116] used the same definition of social media [117].

Overall, the quality of studies was mixed with 41 of moderate or higher methodological quality (strong 17; satisfactory 10; moderate 18) with 21 being of fair (17) or limited (4) quality, and there were 6 where we were unable to apply a quality framework. Despite a lack of methodological quality for a significant proportion of studies, all were retained in the review because of the limited contemporary evidence base and to therefore provide a comprehensive synthesis of this topic area.

### Social Media Used by Health Care Professionals

Health care professionals currently use a broad range of social media platforms in practice, although understanding the extent is limited by study methods used and a lack of population data. Previous literature reviews [12,110-113,115,116] described use of most social media by most HCP groups to communicate interprofessionally and intraprofessionally and with health care consumers. The common types of social media platforms identified in this review were Listservs (n=22), Twitter (n=18), general social media (n=17), discussion forums (n=7), Web 2.0 (n=3), topic-specific discussion forums plus document repositories (n=3), a wiki (n=1), and Facebook (n=1). Physicians (n=24) in general and from 14 clinical specialties were the most common professional group studied, followed by nurses (n=15) in general and from 9 specialty areas, 4 groups of allied health professionals (n=14), health care professionals in general (n=8), a multidisciplinary clinical specialty area (n=9), and midwives (n=2).

Four papers described the uptake and use based on a population of potential users. Twenty percent or more had joined Listservs for occupational health practitioners [65], nurse practitioners [28], and intensive care (nurse data only) [118]. Although 209 of 1559 (13.07%) Korean emergency physicians had participated on a Facebook page [98], only 1.6% of US and 1.7% of Australian emergency physicians had joined Twitter by 2011 [119].

A number of studies of variable quality evaluated the general use of social media and found that health care professionals reported or demonstrated limited use of social media for professional purposes, and when they did, they preferred specialty-specific closed communities. Only 2 studies however were of a high-to-moderate quality [97,120]. A study examining US physicians’ professional use of social media for connecting with colleagues reported limited use; only 52% currently used closed Web-based communities, 25% used wikis, whereas less than 20% used Facebook, podcasts, blogs, or Twitter. More than half also indicated they were unlikely to use these latter 4 platforms in the future [120]. A mixed-method study [97] used diaries to directly track the use of Web 2.0 by 35 junior physicians; 2.6 medical sites were accessed per day, and 52.9% of these visits were to user-generated platforms, but, there was limited professional use of SNS. A study of a broad range of Australian HCP found limited use and knowledge of Web 2.0 technologies; although the response rate was 9.2% (89/965), there were limited responses by physicians, and the researcher was unable to distribute to nurses [107]. The remaining surveys, of Greek health care professionals [109], pharmacy preceptors [106], mental health [101], family physicians [102], and urologists [99,104] found limited social media use, including social networks, for professional purposes. A single study of limited quality [103] found that 80.0% of respondents were using social media for professional purposes; however, the specific purpose was highly variable with only 44.1% using it for professional networking and 26.9% for obtaining or disseminating research evidence and professional development.

Two theories were applied across 3 studies to understand actual or future use of social media by health care professionals. Two high-quality studies applied the theory of planned behavior; a survey on the future use of Web 2.0 by Hong Kong nurses [121], and a qualitative study on the use of a wiki to transfer best practice care for patients with head injuries, where nurses were considered credible or influential peers by physicians [122]. Another survey of US physicians [120] applied the technology acceptance model (explains human behavior in relation to computer use) to explore user adoption. To use social media, clinicians required a positive attitude that the media was easy to use (usability), they were able to have a practice run to see how it worked (trialability), the platform worked better than current solutions (relative advantage), and the technology was accessible in the workplace and fitted in with current work practices. The final mediating factor was that their peers also shared these attitudes, a reflection of the influence of homophily.

### Social Media and Virtual Communities

Overall, 36 reports described 31 discrete virtual communities [27,31,65-69,71,74,76,79,81-84,88,92,94-96,98,100,105,118,123-126] that were established in 3 main ways. The most common were discussion forums or Listservs created by a professional society [28,65-67,69-71,74,76,79,83,91,94,98,105,123]. Nine communities appear to be have been established by an individual or group of HCP using inexpensive or open access platforms such as Yahoo groups, mailing list software, or Twitter [27,68,81,84,88,92,95,124,127]. Eight communities were established by a government health department with the purpose of improving communication and knowledge distribution between health care professionals to enhance care [31,82,96,100,118, 125,126,128,129].

The most common reasons for establishing a discrete virtual community were to:

1. Create a professional forum where relevant professional and academic issues could be discussed and information and knowledge shared [28,67,69,70,73,76,79,91,95,98,100, 105,125,127-130].

2. Address professional isolation [70,73,91,105, 126-128].

3. Facilitate networking [27,76,83,91,100,105,124,127,128].

4. Foster peer collaboration and mentoring [83,100,105,128].

5. Facilitate professional development [74,83,91].

6. Improve clinical practice through research and evidence translation [100,127].

7. Obtain clinical advice or opinion [98].

Where a distinct professional community was evaluated, 31 of 36 were a virtual community in a single HCP discipline such as physician, nurse, occupational therapist, social worker, pharmacist, or medical librarian. Note that these virtual communities were for a specific clinical specialty, except for 2 nursing communities (Nursenet [81,92] and allnurses [68]) and the medical librarian virtual community [73,94]. Five multidisciplinary virtual communities were all Listservs for a clinical specialty established by: (1) an international professional society for travel medicine clinicians [71]; (2) a Norwegian professional society for occupational health clinicians [65]; (3) Spanish speaking radiation medicine clinicians [66]; (4) an Australian jurisdiction–based health unit for intensive care clinicians [118,126] and a Twitter network connecting physicians from 3 specialty areas [131].

Social network analysis of 3 virtual CoPs demonstrated early evidence supporting the flow of knowledge across virtual communities. A study examining the growth and social network of an intensive care Listserv demonstrated an evolution from a single-state nurse-specific network to an Australian-wide, multidisciplinary, and multiorganizational network over 6 years [118]. A distinct Twitter virtual community, created via following patterns of emergency physicians (board certified in United States or Australia) showed a small core (2.8%) with a larger interconnected group, although 34% were not connected to any others [119]. Another study examined Twitter virtual community connections across 4 physician groups from the United States and reported 4 distinct communities with a small overlap where there was some information flow between groups [131].

The question of whether a CoP might be possible using social media was examined in several studies. Three high-quality qualitative studies exploring a critical care nursing Listserv found that motivators of Web-based knowledge sharing mapped to key aspects of CoP theory, including reciprocity, collectivism, respectful environment, and altruism [27,88,124]. A survey of a nurse practitioner Listserv reported that a sense of community correlated with learning (Pearson coefficient = +.94) and connectedness (r=.95), although the response rate was only 22%, and there was no indication whether respondents were active posters or nonposters [28]. A literature review [12] adapted a 7-item framework for a health care virtual CoP from a business model [132], exploring: (1) facilitation; (2) champion and support; (3) objectives and goals; (4) a broad church; (5) supportive environment; (6) measurement, benchmarking, and feedback; and (7) technology and community.

### How Members Use Social Media Virtual Communities

Most research on how health care virtual communities were used by members focused on posting behaviors. Web-based roles of members can be broadly described as participants (Web-based posters) and nonposters. Direct measurement of posting behaviors across a number of platforms demonstrated a pattern of a minority of members being responsible for most posts [31,65-67,69,82,94,130] or conference tweeting [77,86,87,89,90] (see Table 1; Multimedia Appendix 6). The same pattern was revealed across 4 surveys asking health care professionals about their Web-based behavior [69,94,96,126].

**Table 1:** Summary of studies examining Web-based posting behaviors by virtual community members.

Reference	Social media; time span	Nonposting	Low posting	Medium posting	High posting
Cervantez Thompson [76]	Listserv; 18 months	33%	27.8% at least once in 18 months		10 members > 30
Long et al [70]	Discussion forum; 12 months	28.3% (n=170)	48% (n=239) < 4 times	30% (n=179) 4-20 times	0.2% (n=12) 19-59 times (17% of total data corpus)
Stewart et al [125]	Discussion forum; 27 months	33% (n=14)	46% (n=21) < 14 times	13% (b=6) 15-28 times	9% (n=5) 29-56 times
Rodriguez-Recio and Sendra-Portero [66]	Listserv; 5 years	46.3% (n=175)	434% (n=161) 1-10 times	8% (n=30) 11-30 times	3.2% (n=12) 31 to < 200 times
Macdonald et al [71]	Listserv; 6 months				Top 20 users—43% of posts
Morken et al [65]	Listserv; 1997-2004		Average number of posts = 2.1; this reduced to 0.6 in 2004		
Brooks and Scott [31,82]	Discussion forum		11 aged care nurses posted over 7 months	26 cardiac nurses posted over 7 months	29 midwives posted over 1.5 months

Conversely, “non-posting” or “lurking” behavior [133] was generally high, ranging from 28% to 46% (see Table 2). These findings however do not indicate whether nonposters were active in reading posts. Where being active nonposters was directly measured, it ranged from 1% to 33%, whereas survey respondents self-reported reading levels of post as 64% to 96% (see Table 2).

**Table 2:** Summary of studies examining reading (access) behaviors.

Reading	Social media	0	Low	Medium	High
Stewart and Abidi [125]	Discussion forum; Web-based observation; access	32.6%	54.3%	4.3%	8.7%
Cook-Craig and Sabah [96]	Discussion forum; Web-based observation; access	1%	11%	38%	50%
Rolls et al [126]	Listserv; survey		3.5%	13.2%	83%
Schoch and Shooshan [94]	Listserv; survey; access		36%	24%	40%
Kim et al [98]	Facebook; survey; access	once or less each week—22.3	2-4 times per week—23.7%	5-6 times per week—16.6%	> 1 per day—37.4%
Whitaker et al [69]	Listserv; survey	Seldom or never 10%	1 per week to month	Several times per week 40%	Daily 40%

Current evidence describing barriers and motivators to posting over the Internet is difficult to quantify; only 4 studies examined these elements, 2 of which reviewed the same Listserv and included frequent poster activity [27,88] (see Table 3). These limited data suggest a symbiotic relationship between members and the Web-based community, with behaviors of posters influenced by both access to new knowledge and contributing for other members of the community. These elements of altruism, reciprocity, and collectivism are essential components of CoP building [27,28,88]. Reported barriers suggest that knowledge self-efficacy and time are key mediators of Web-based participation or knowledge sharing in health care virtual communities [88,126].

**Table 3:** Mediators of posting in by health care professionals in social media and virtual communities.

	Individual level	Community level
Motivators	Personal gain: (1) more knowledge [28,77,88]; (2) a better reputation [88]; (3) emotional support [88]	Collectivism [28,77,88,124]
	Seeker interest [88]	Reciprocity [28,88,124]
	Altruism [28,77,88,124]	Respectful environment [88,124]
	Self-selection [27]	Technology [88,124]
	Validation of one’s practice [27]	Asynchronous nature [27]
	Advocacy [77]	Facilitate networking [77]
	Better understanding of current knowledge and best practice in the field [27,77,103]	Noncompetitive environment [27]
Barriers	Nothing to add [88]	Information not trustworthy [103]
	Nothing to say [126]	Lack of privacy [28,103]
	Lack of time [88,103,126]	Technology [88,103]
	Unfamiliarity with subject [88]	Confidentiality of sharing organization documents [88]
	Lack of confidence [126]	Tone of discussion [28,126]
	Local unit constraints [126]	Alienation[28]
	Attitude of seeker [88] or poster agenda[103]	Unprofessional behavior [28,103]

Overall, these findings supported the use of social media by health care professionals, specifically discussion forums and mailing lists platforms, to develop virtual professional CoPs. These communities valued the Web-based forums as information or knowledge portals, enabling members to “keep up to date” [73,94,124] with clinically relevant and quality information [66], develop workplace resources [123] and benchmark practice [27,123,124]. Importantly, access to a broader range of professional colleagues beyond their local organization enabled members to make more informed practice decisions, with greater confidence that these decisions reflected current best practice [124].

### Manifest Content of Posts

Manifest content is the text immediately visible and easy to identify and count [61]. The quality of evidence describing the manifest content of posts, including posting behaviors, number of posts, length of discussion thread, and ratio of initial post to responses, was limited by both the quality of studies (See Multimedia Appendix 7) and variability in the sampling and measurement methods used. Making sense of the types of posts in social media was also challenging as researchers used variable descriptors when categorizing post types. The proportion of clinical versus nonclinical posts varied greatly across studies. Clinical posts were in the majority across 5 Listservs: travel medicine professionals (88%) [71]; radiology professionals (71.8%) [66]; rehabilitation nurses (60%) [76]; forensic occupational therapists (59.9%) [127]; and occupational health (54%) [65]. Posts on professional issues were more common on a plastic surgery discussion forum (60% concerned education and introduction of new members) [79] and an international nursing discussion forum (83% focused on career and education advice, work issues, and handling job-related emotions) [68]. Analyzing categories of conference tweets revealed similar results to Listserv and discussion forum data; however, understanding how it related to clinical knowledge or new research was difficult because of variable taxonomies and mixed quality. Five studies, evaluating 8 conferences, used the same taxonomy [134] and found that tweets concerning conference content (termed informative) ranged between 20% and 30% [78,87], 30% and 40% [78,135], 40% and 50% [78,136], and 50% and 60% [78]). Similar data were found across 2 conference years where most tweets from an oncology conference were clinical topics (54.5% and 60.4%), such as clinical management discussions and clinical news or trial outcome [80]. Contrasted against this was a study of an emergency conference, which found that 75% of tweets related to conference content [90]. Note however that the most commonly used taxonomy [134] has limited validity within or generalizability to health care conference data, as it was developed from a single Twitter feed specific to the author, it was not reviewed by a second coder or tested against another dataset. A systematic review of Twitter journal clubs that cross-referenced hashtag use with Web-based data [114] found sustained and increasing use of 5 specific tags (#ADC_JC; #ebnjc; #IGSJC; #Nephjc; and #urojc).

Four studies of mixed quality found that topics of clinical posts in virtual communities mapped to the knowledge domain of a professional specialty. Within a travel medicine Listserv, there were 27 topics across 5 major categories (vaccine preventable diseases, vector-borne diseases, pretravel, general, and miscellaneous) [71]. Pediatric occupational therapists posted on 4 categories (practice, performance component, performance area, and health conditions) [70]. Members of an occupational health forum posted on 4 clinical categories (chemical hazards, methods in health and safety environment, ergonomics, and noise and radiation) [65]. Pharmacists discussed a broad range of topics including patient and clinical problems, pharmacy politics, legal issues, drug tariffs, government policy, business and finance, risk management, and pharmacy information technology [69].

### Latent Content of Posts

Latent content reflects the hidden meaning of textual content by a researcher [137]. Latent content examined included types of knowledge exchanged and presence of discussion and existence of knowledge work. Understanding the types of posts was limited by variability in study methods and challenging because of widely varying definitions and lack of robustly developed content analysis tools. Only 3 studies examined the types of knowledge within virtual community posts (Multimedia Appendix 6). Two high-quality studies that examined a nursing Listserv found that more than 90% of knowledge exchanged was practical knowledge (related to institutional practices, personal opinion, or suggestion) rather than book knowledge (facts, general regulations, statutes, or published works) [27,88]. On a Spanish radiological Listserv, 43% of emails were classified as scientific information [66].

As described earlier, knowledge work involves elements of interaction, critical reflection, and learning as a dialogical process [31,138]. Only limited data were identified supporting the presence of knowledge work within virtual professional communities. Three studies [66,73,130] described the presence of discussion or meta-discussion within emails exchanged; however, no content analysis tool or definitions were provided to justify these conclusions. One single high-quality study [81] effectively described the presence of reflection in discussion, where participants reported changes in practice through an iterative process that included off-line and Web-based discussions. One organizational project demonstrated mixed results, with high levels of knowledge work on a midwifery forum but lower levels in both aged care and cardiology forums [31,82,139].

## Discussion

The focus of this review was to identify whether health care professionals have effectively created virtual communities to facilitate professional networking, knowledge sharing, and evidence-informed practice. The current evidence is mixed in terms of quality and type of studies undertaken. Apart from a couple of exceptions, studies published before 2004 were limited by common methodological limitations including sample and measurement bias, especially when content analysis techniques or surveys were used. The quality of more recent studies, including those using focus groups, surveys, interviews and Q-sort, has improved and reveals important insights into how health care professionals use social media to develop virtual communities and interact with professional colleagues. Importantly, these insights indicate that virtual communities may provide significant opportunities to overcome current barriers to knowledge flow and professional networking in health care.

This beginning evidence supports the view that health care professionals have adopted social media to create viable virtual professional communities, and that health care virtual communities share similar characteristics to other professional communities. A consistent pattern in Web-based communities was that most contributions were attributed to a limited number of individuals [31,65-67,71,76,82,130]. The voluntary nature of participation within social networks and virtual communities means that members participate at different levels and may adopt specific Web-based roles [140]. A virtual community is likely to have a mixture of lurkers, observers, passive, and active contributors [141]. Importantly, nonposting virtual community members continue to belong because of potential access to important information (reflective of Burnett’s information neighborhood) [142], but, this requires further investigation.

There is a modest level of evidence that the most common activity in health care virtual communities is the exchange of experiential domain-specific knowledge. Importantly, the rise of conference tweeting and journal clubs suggests that Twitter may have a role in reducing the evidence practice gap. There are however only limited contemporary data supporting the transfer of empirical knowledge or how this new knowledge is used in practice [27,80,87,123,124]. In addition, although there are generally positive attitudes toward and intention to use social media [120,121], a skepticism persists regarding the veracity of information [97,103,122]. Understanding the exchange of knowledge remains limited as all but one study [77] failed to appreciate that social media interactions reflect a conversation with each post likely influenced by an antecedent [143].

Gaining access to previously unknown information or knowledge is an essential benefit of networking [20], and sharing this information is a major driver of social networks and virtual communities [144]. Effective knowledge transfer and innovation development occurs in social networks where there is a shared understanding of knowledge but also a density of ties providing access to novel information [20]. The symbiotic relationship between the culture of a virtual community and its members creates an ethos of knowledge sharing in a Web-based context. Similar to nonhealth virtual community [145,146], Web-based knowledge sharing is facilitated by a culture of altruism, trust, collectivism and reciprocity, as well as a respectful noncompetitive environment [28,88,124,126]. Knowledge self-efficacy, a belief the answer supplied is correct and worthwhile, influences knowledge sharing by individuals [147-150]. Moreover, group behaviors perceived as negative (eg, tone of discussion or contentious issues) have an undesirable effect on both willingness to share knowledge and retention of community members [28,126,142].

The dominance of Listservs and discussion forums in this search period is not surprising, given these platforms have been available since the early 1990s [48]. Although these social media platforms provide HCP with the ability to interact, they are limited in functionality, particularly with their capacity to create and/or store permanent community artifacts (such as guidelines or learning packages) required by a CoP for knowledge and practice development [4]. The relatively recent arrival of Web 2.0 platforms, enabling users to create and/or upload content, overcomes these problems [47]; however, there were only 2 reports [105,128] of virtual communities using this modality evident in this review. Conference tweeting, tweet chats, and journal clubs haves emerged in recent years; however, the current variability in methods used limits our understanding how this might contribute to distribution of scientific knowledge.

At this time, the evidence suggests that clinicians prefer to use social media that allows them to communicate within their own profession and within a clinical specialty, as most virtual communities identified were for a clinical specialty within a single HCP discipline. Although this may reflect continuing tribal behavior of clinicians in practice [23,24,151], monodiscipline social networks can create strong boundaries that inhibit interprofessional learning and knowledge sharing [152] and promoting practice initiatives to improve patient outcomes [151]. Sharing knowledge and adoption of innovation is enhanced where there is homophily (shared within a multidisciplinary clinical specialty domain such as emergency or intensive care) and credibility [152]. Because patients are commonly cared for by a multidisciplinary team and these clinicians generally share a common specialty knowledge domain, multidisciplinary networks are more likely to be effective in knowledge transfer and creation [20,42]. In this review, this potential was demonstrated in 2 multidisciplinary virtual communities [118,125]. A social medium that creates an open virtual community through user-generated follow patterns (such as Twitter) has this potential, but this is yet to be demonstrated in health care.

### Strengths and Limitations of the Review

The key strengths of this review were the timeline, promoting the inclusion of the broad range of current social media apps, and the specific focus on voluntary professional participation. Previous reviews were unable to provide clear information on our focused question because of inclusion of education and undergraduates [110,115] or patients [116]. Nonetheless, exclusion of research within a training framework remains a limitation as does the exclusion of wikis and other collaborative writing technologies and blogs. Another limitation was the keyword search, where we were dependent on how keywords were applied when papers were published. Of note, the term social media was only added to the MeSH list in PubMed in 2012. We attempted to address this by undertaking a series of searches (see Multimedia Appendix 1) using a range of keywords; however, we may not have captured all relevant publications. Moreover, we only used English language publications, so we may have missed other important studies.

### Recommendations for Further Research

As the current evidence is limited in quality and with most studies examining older technological platforms, there are a number of recommendations for future research. Recent studies [65,70,71] show that solicitation and supply of knowledge of craft-specific knowledge are the most common posts exchanged on professional health care virtual communities. There are limited data however to describe: (1) the specific types of knowledge exchanged (eg, scientific vs experiential or tacit vs explicit); (2) accuracy of this knowledge; (3) whether the knowledge supplied addressed what the poster requested; and (4) what the receivers of the emails, including the original poster, did with this knowledge. Further content analysis of posts using a more systematic approach may reveal not only the knowledge needs of members but also the knowledge embodied within the network.

At present, there is limited understanding of why individuals join or participate in a Web-based community; previous studies have generally examined activity from the perspective of Web-based posters. Some data suggest that professionals will join a virtual community where they find local resources inadequate [153]. Importantly, although nonposters or limited posters constitute a large portion of virtual community membership, it is not clear why they belong to the community or why they chose to limit posting. Because movement of knowledge or innovation into and around an organization is the role of boundary spanners and knowledge brokers (eg, educators or researchers), do these individuals see membership as a valuable tool for their substantive position, as preliminary data suggest [123]? If so, could health care organizations improve knowledge flow by facilitating communication between key personnel using Web-based communities? Understanding these phenomena is important if leaders or moderators of virtual communities, researchers, or health system change agents are to create optimal Web-based experiences and ensure the viability of the social medium within professional health care environments.

Early research suggested that Web-based forums may facilitate the development of higher order cognitive skills, such as tertiary students’ critical thinking [154]. These important findings may be linked to educational design, implementation, and evaluation for effective adult learning by today’s HCP. This contrasts with the self-selective and voluntary nature of professional forum membership. Only 2 studies verified the presence of a CoP within a Web-based health care community [28,124]. There is however now a worldwide education movement based around the use of social media for the professional development of clinicians. Free Web-based medical education (#FOAMed) [155] is an egalitarian movement promoting open Web-based publication of a wide range of resources for the education of any clinician. Further research is required however to identify the viability of social media platforms for voluntary professional development of health care professionals. This may require a mixed-methods approach to comprehensively understand the learning interaction (via a social network analysis), process (via content analysis), and outcome (via a survey) [156].

### Conclusion

The current evidence on the use of social media by health care professionals suggests that virtual communities are viewed as valuable knowledge portals where craft knowledge is exchanged. This review, apart from the recent emergence of conference tweeting and Twitter journal clubs, found only a limited number of publications concerning newer social media platforms. Arguably, the current range of social media platforms and electronic devices facilitating exchange of information makes professional networking possible wherever the Internet is available. Given that a number of the current challenges of TRIP are related to a lack of inter professional and intraprofessional communication channels, there is significant potential within multidisciplinary virtual communities to facilitate the transfer of experiential and research knowledge by breaking down professional and organizational boundaries. Further research is required to evaluate whether virtual communities may improve patient outcomes by facilitating professional development, evidence-based practice, and elimination of clinical practice silos.

## Appendix

Multimedia Appendix 1 Detailed search strategy and results.

Multimedia Appendix 2 Overview of all studies included in final review.

Multimedia Appendix 3 Quality assessment table for qualitative studies.

Multimedia Appendix 4 Quality assessment of studies using content analysis techniques.

Multimedia Appendix 5 Quality checklist for questionnaire surveys.

Multimedia Appendix 6 Quality assessment of reviews.

Multimedia Appendix 7 Content of posts on health care social media.

## Abbreviations

CoP: community of practice
HCP: health care professionals
SM: social media
SNS: social networking sites
TRIP: translating research into practice
VC: virtual community
