# Supplementary material for: How Health Care Professionals Use Social Media to Create Virtual Communities: An Integrative Review
Source: J Med Internet Res. 2016 Jun 16;18(6):e166. doi: 10.2196/jmir.5312 (PMC4933801; doi:10.2196/jmir.5312)
Supplement: Multimedia Appendix 1 [file jmir_v18i6e166_app1.pdf]

Database	Key word OR word in abstract where not a MESH term in database	limits	years	hits	cull	repeats	reviewed
CINAHL1	computer mediated communication	abstract available<br>English<br>research article<br>peer-reviewed	1/1/1990-31/12/2015	51	7	0	7
CINAHL2	listserv	abstract available<br>English<br>research article<br>peer-reviewed	1/1/1990-31/12/2015	110	17	1	16
CINAHL3	online discussion forum	abstract available<br>English<br>research article<br>peer-reviewed	1/1/1990-31/12/2015	25	3	1	2
CINAHL4	networking or social networking	abstract available<br>English<br>research article<br>peer-reviewed	1/1/1990-31/12/2015	373	14	9	7
CINAHL5	discussion forum OR twitter OR social media OR Facebook	abstract available<br>English<br>research article<br>peer-reviewed	1/1/1990-31/12/2015	459	14	1	13
CINAHL6	virtual community	abstract available<br>English<br>research article<br>peer-reviewed	1/1/1990-31/12/2015	81	15	13	2
CINAHL7	social media	abstract available<br>English<br>research article<br>peer-reviewed	1/1/1990-31/12/2015	97	2	2	2
pubmed1	social media	abstract available<br>English<br>research article<br>peer-reviewed	1/1/1990-31/12/2015	677	32	3	25
pubmed2	social networking AND doctor or nurse or pharmacist or respiratory therapist or pharmacist or social worker or dietitian	abstract available<br>English<br>research article<br>peer-reviewed	1/1/1990-31/12/2015	305	11	5	6

Database	Key word OR word in abstract where not a MESH term in database	limits	years	hits	cull	repeats	reviewed
pubmed3	electronic mail AND doctor or nurse or pharmacist or respiratory therapist or pharmacist or social worker or dietitian	abstract available<br>English<br>research article<br>peer-reviewed	1/1/1990-31/12/2015	447	3	0	3
pubmed4	virtual community AND doctor or nurse or pharmacist or respiratory therapist or pharmacist or social worker or dietitian	abstract available<br>English<br>research article<br>peer-reviewed	1/1/1990-31/12/2015	132	10	3	7
pubmed5	online discussion forum AND doctor or nurse or pharmacist or respiratory therapist or pharmacist or social worker or dietitian	abstract available<br>English<br>research article<br>peer-reviewed	1/1/1990-31/12/2015	192	7	2	5
Journal search	JMIR - Journal of medical and internet research	abstract available<br>English<br>research article<br>peer-reviewed	1/1/1990-31/12/2015	12	9	8	1
	JAMIA - Journal of medical informatics association	abstract available<br>English<br>research article<br>peer-reviewed	1/1/1990-31/12/2015	3	3	2	1
	CIN - Computers, Informatics, Nursing	abstract available<br>English<br>research article<br>peer-reviewed	1/1/1990-31/12/2015	4	4	3	1
Proquest Health & Medicine	social media; mailing list; discussion forum			2	2	1	1
				2970	153	54	99
						quality	5
						not on topic	26
						cull	31
						from journal articles	4
						total in review	72
