# Supplementary material for: How Health Care Professionals Use Social Media to Create Virtual Communities: An Integrative Review
Source: J Med Internet Res. 2016 Jun 16;18(6):e166. doi: 10.2196/jmir.5312 (PMC4933801; doi:10.2196/jmir.5312)
Supplement: Multimedia Appendix 2 [file jmir_v18i6e166_app2.pdf]

Author/country/Social media type	Purpose	Design	Sample/data corpus	Data collection	Data analysis	Quality
Berman 1996<br>Israel<br>Social workers<br>Listserv[1]	Study the potential of people as sources of information within an information technology framework, the Internet	Qualitative descriptive	Data corpus<br>▪ SOCWORK – 37 days<br>▪ ABUSE-L – 43 days	Online observation using Empireer Classification:<br>▪ Information transfer – IT<br>▪ Information request – IR<br>▪ Discussion of issues - IS	Content analysis - deductive	Fair
Murray 1996<br>International<br>Nurses<br>Mailing list – internet based[2]	Investigate the use of computer mediated communication technologies by nurses	Case study using mixed methods	2 days emails<br>5 self-selected	Online observation<br>Interviews Email self selected	Discourse analysis	Moderate
Schoch 1997<br>United States/international<br>Mailing list<br>Medical librarians[3]	Determine demographic characteristics and use of mailing list	Survey	Random sample	Survey Electronic	Descriptive	Moderate
Roberts 1998<br>United Kingdom<br>General Practitioners<br>Listserv[4]	Explore the dynamics of internet based discussion group	Qualitative - ethnography	12 months emails	Online Observation	thematic	Fair
Murray 2001<br>International Nurses<br>Mailing list – internet based[5]	Examine whether there is evidence of reflection, and outputs of reflection (such as learning and changes in practice) arise through discussions on a mailing list	Online ethnography	Survey 1 – random sample Survey 2 – all members<br>Email data corpus<br>▪ 2days (1994-2000)<br>▪ Stratified-purposive sample<br>○ DT > 4 messages<br>○ Identifies clinical	Member surveys (2) stratified<br>Online observation	Content analysis - inductive	strong

Author/country/Social media type	Purpose	Design	Sample/data corpus	Data collection	Data analysis	Quality
			practice issue			
Reutzel 2001<br>United states<br>School nurses<br>mailing list[6]	Obtain a preliminary understanding of the types of medication management problems that school nurses face as well as the strategies they use to solve those problems	Qualitative descriptive<br>Content analysis of emails – deductive	Data corpus - 5/12 emails<br>stratified sample<br>Unit of analysis – Discussion thread focusing on medication issues	Coding schema – 7 categories with additional 3 arising	Content analysis - deductive	strong
Cervantez-Thompson 2002<br>United states<br>Rehabilitation nurses<br>Listserv<br>[7]	Identify the profile, postings and roles of nurses on a mailing list	Qualitative descriptive	Data corpus all postings May 1999-Nov 2000<br>Unit of analysis – individual emails	Online Observation<br>Census sampling	Content analysis - deductive	Fair
Watson 2003<br>Australia<br>Infectious disease specialists<br>Mailing list[8]	Determine level of user satisfaction with mailing list	Survey	All members of listserv	Not described	descriptive	fair
Cervantez-Thompson 2004<br>United states<br>Rehabilitation nurses<br>Mailing list[9]	Why members use mailing list and describe their experience	Mixed methods survey + interviews	▪ Purpose sample – online posters<br>▪ Response rate 22% (76/343)<br>Interviews – 41/76 self-nominated from survey 1 responses	1. Online questionnaire – 5 open questions<br>2. Follow up telephone or email interviews	Survey – descriptive<br>Interviews – grounded theory	moderate
Brooks 2006<br>United Kingdom<br>Midwives<br>Intranet -discussion	To evaluate whether midwives would function as knowledge workers in an online	Mixed methods - Case study<br>1. Content analysis – thematic	2. Data corpus – 3/12 posts<br>Interviews – 15 online participants (purposive	Online observation<br>Discussion forum posts<br>Interviews (face to face and semi structured)	Discussion forum<br>3. Themes – knowledge work and relationships	strong

Author/country/Social media type	Purpose	Design	Sample/data corpus	Data collection	Data analysis	Quality
forum[10]	forum	2. Participant Interviews	stratified sampling)		(collegial and leadership)<br>4. Midwifery grade of poster	
Brooks 2006<br>United Kingdom<br>Midwives<br>Nurses<br>Intranet -discussion forum[11]	Explore the level of knowledge work displayed in three intranet based discussion forums	Mixed methods<br>Intranet based discussion forums<br>- Obstetric (Obs)<br>- Older persons (OP)<br>Coronary heart disease (CHD)	Data corpus<br>1. Obs - 1.5/12<br>▪ 29 posters / 11 threads / 70 , posts<br>2. OP - 7.5/12<br>▪ 11 posters / 6 threads / 18 messages<br>3. CHD - 15/12<br>1. 26 posters / 21 threads / 71 messages	Census sampling<br>Knowledge work taxonomy (12 item framework)<br>Semi structured interviews	DT - descriptive<br>Interviews - grounded theory (Atlas.ti)	strong
Hara 2007<br>International<br>Advanced practice nurses (critical care)<br>Mailing list[12]	Examine<br>1. the types of online activity<br>2. types of knowledge shared<br>3. factors that sustain knowledge sharing	Case study using mixed methods<br>Triangulation<br>CoP theoretical framework	Emails<br>1. Data corpus – Weeks 1 & 2 of each month 2005<br>2. Unit of analysis<br>a. Knowledge – email<br>b. Online activity - thematic unit<br>Interviews – semi-structured -27	Online observation<br>Interviews	1. types of knowledge - content analysis<br>2. Types of online activities -Constant comparative<br>3. Factors that influence knowledge sharing – constant comparative	Strong
Hew 2007<br>International<br>3 mailing list<br>1. Advanced practice nurses [APN-l]<br>2. University web development	Categorize the types of knowledge shared<br>Identify the motivators of and barriers to online knowledge sharing	Mixed methods – comparative case study	Data corpus – weeks 1& 2<br>1. 3/2003-2006<br>2. 1/2003-2006<br>3. 2/2003 -2006<br>Unit of analysis – thematic unit	Online observation – types of knowledge shared<br>Semi-structured telephone Interviews – motivators & barriers to knowledge sharing	Emails – content analysis – deductive<br>Interviews – constant-comparative	Strong

Author/country/Social media type	Purpose	Design	Sample/data corpus	Data collection	Data analysis	Quality
[WD-l]<br>Literacy education [LE-l] [13]<br>3.			Interviews – semi-structured<br>1. 16<br>2. 18<br>3. 20			
Rodriguez-Recio 2007<br>Spain<br>Radiology clinicians<br>Mailing list[14]	Analyse mailing list during first 5 years of operation including content of posts and perception of members	Mixed methods<br>▪ Content analysis (deductive)<br>▪ Member survey<br>▪ Social network analysis	• Data corpus – 5 years<br>• Survey<br>○ Demographics<br>○ Reading patterns<br>○ Listserv management<br>○ Networking ex-listserv<br>○ Evaluation of reading list (functionality, usefulness and quality of email content	1. Online Observation<br>2. survey anonymous & online<br>3. Social network analysis	Descriptive<br>Inferential	fair-moderate
Hew 2008<br>International<br>Advanced practice nurses (critical care)<br>Mailing list[15]	Gain an understanding of knowledge sharing among nurses on a mailing list	Qualitative	Round 1 – 27<br>Round 2 – 10 most frequent online knowledge sharers of round 1	Semi-structured telephone interviews – 2 rounds	Constant comparative<br>Baston – motivational theory	Strong
Rolls 2008<br>Australia<br>Intensive care<br>Mailing list[16]	Explore the perceptions of members of mailing list	Mixed methods	Email data corpus – 6/2004-5/2005<br>Instrument – 25 item (piloted)	Online observation<br>Survey	Descriptive<br>Content analysis - deductive	Moderate
Widemark 2008<br>Arizona – US	Evaluate the effectiveness of leaning	Mixed methods<br>- Survey - all	Survey 1 – 650 – 146<br>Response rate -22%	Survey 1 – Classroom community scale	Survey 1 – quantitative with	Survey - Mod-strong

Author/country/Social media type	Purpose	Design	Sample/data corpus	Data collection	Data analysis	Quality
Nurse Practitioners<br>Mailing list–closed[17]	in a situated learning environment	- Qualitative survey 10	convenience<br>Survey 2 – 8/10 convenience	20 items/5 point likert<br>Survey 2 – 12 questions<br>6 demographic<br>6 open	correlation/regression analysis<br>Survey 2 – inductive content analysis	Qualitative – Fair
Cook-Craig 2009<br>Social workers<br>Israeli ministry [18]	Explore how social workers in Israel use virtual community of practices (VCoP) to support learning	Mixed methods<br>- Quantitative<br>- Survey	All online participation<br>Survey – random sample 300 (Response rate 33%)	VCoP usage data<br>Online Survey – 35 item		Admin data – moderate<br>Survey - fair
Hughes 2009<br>UK<br>Physicians<br>Web 2.0 [19]	Examine the use of Web 2.0 by junior physicians in clinical setting including motivations, direct use & how can tools be further used	Mixed methods<br>Diaries<br>Interviews	35 junior physicians<br>177 diaries days	Diaries<br>Interviews	Thematic analysis	Moderate
Long 2009<br>Australia Paediatric occupational therapists<br>Listserv - internet[20]	Gain insight into the nature of communications o mailing list and determine whether topics and issues were congruent with current practice trends	Qualitative descriptive	Data corpus – 6/2003 – 5/2004	Content analysis deductive<br>Coding schema – OT curricula<br>Census sampling	Content analysis - deductive	Moderate
Macdonald 2009<br>International<br>Listserv – professional society<br>Travel medicine[21]	Analyse<br>• patterns of information exchange on mailing list<br>• subscriber demographics<br>• participation rates	Qualitative descriptive	Data corpus – all emails 1/2006-/7/2006	Online observation<br>Census sampling	Content analysis - deductive	Moderate
Morken 2009	Describe the activity on	Qualitative descriptive	○ Data corpus –	Online Observation	Content analysis -	Fair-

Author/country/Social media type	Purpose	Design	Sample/data corpus	Data collection	Data analysis	Quality
Norway<br>Occupational hygienists Listserv [internet] [22]	a mailing list		categorized all emails 1997-2006<br>○ Data corpus –2006 emails<br>▪ By discipline<br>▪ Type of email<br>▪ Completeness of online answer	Census sampling	inductive	moderate
Shanahan 2009<br>Australia<br>Medical radiation specialist<br>Internet based tools[23]	Establish professional use of internet-based tools by clinician and issues affecting access to Internet within the workplace	Qualitative	Random sample of medical radiation science practitioners	Survey	Descriptive	Moderate
Foong 2010<br>India<br>Plastic surgeons<br>Discussion forum[24]	To assess the value of discussions in relation to education and aiding patient management	Qualitative descriptive	Calendar year	Online observation census	Deductive content analysis	fair
Franko 2011<br>USA<br>Orthopedics surgeons<br>Twitter [25]	Analyse the type and prevalence of orthopedic surgery-related profiles on Twitter in regard to self-identified surgeons	Qualitative descriptive	All identified as orthopedic	Online observation	descriptive	NA
Hoffman 2011<br>Australia - QLD<br>OT<br>Website [26]	Explore occupational therapists perceptions of the benefits of, barriers to and reason for using or not using an online CoP	Mixed methods<br>- Focus groups<br>- Survey	FG – at national conference	Focus groups (n=2; user/nonuser)<br>Survey (55/673	FG – qualitative descriptive; member checking; thematic analysis (blinded to origin of transcripts	Moderate
Kukreja 2011	To define the current	Qualitative	Convenience sample of	Survey	Descriptive	Moderate

Author/country/Social media type	Purpose	Design	Sample/data corpus	Data collection	Data analysis	Quality
USA<br>Pharmacist<br>Twitter<br>facebook[27]	use patterns of Facebook and twitter among pharmacy preceptors and assess perceptions regarding use of social media within professional practice		pharmacy preceptors	27 item instrument – piloted		
Lau 2011<br>Hong Kong<br>Nurses<br>Web 2.0[28]	to investigate how Web 2.0 tools can be applied for knowledge sharing leaning, social interaction and production of collective intelligence in the nursing domain and to investigate what behavioral perceptions are involved in the adoption of Web 2.0 by nurses	Qualitative	377 Registered nurses working in public hospitals in Hong Kong	Survey<br>- Decomposed theory of human behaviour (DTPB);	Pearson's correlation coefficient, r, and t test	Strong
Valaitis 2011<br>Canada<br>Nurses<br>VCoP[29]	Explore community health nurses' viewpoints on whether a VCoP supported their practice	Qualitative	Statements 66 →44<br>Q-sort – 16 (10% members? Say n= 114 for membership)	Stage 1 – initial statements gathered using online survey (n=15) & focus groups (n=21)<br>Stage 2 – statement refined<br>Stage 3 - Q-sort 16 (following pretesting)	PQMethod 2.11, by-person factor analysis to identify participants with similar points of view<br>Factor extraction – centroid method	Strong
Apostolakis 2012<br>Greece<br>Social media[30]	Level of knowledge and use of internet and social media	qualitative	Greek healthcare professionals graduates of single institution	Survey<br>41 item instrument<br>Piloted		Fair

Author/country/Social media type	Purpose	Design	Sample/data corpus	Data collection	Data analysis	Quality
	technologies acceptance and trust of social media for social, professional and general activities			Cronbach α 0.738		
Archambault 2012<br>Canada<br>Emergency speciality<br>Wiki[31]	To explore participants beliefs on the utility of wiki based reminder regarding best practice management of severe traumatic brain injury	Qualitative	3 sites<br>25 emergency physicians<br>25 allied health	Semi structured interviews	Content analysis – deductive Coding based on Theory of planned behaviour	Strong
Burg 2012<br>USA<br>Social workers<br>Mailing list [32]	to describe the general categories and themes of postings ; examine the process of facilitation of mutual support and information exchange among oncology social workers (OSW)	Qualitative descriptive	Dec 2010-Nov2011	Online observation of listserv	Content analysis - inductive	Strong
Chaudhry 2012<br>USA<br>Oncology physicians<br>Twitter[33]	Explore how Twitter use had expanded over time	Qualitative descriptive	Census sample	Online observation<br>Stratified	Deductive content analysis<br>3 coders – independent	Moderate
Desai 2012<br>Twitter – conference<br>Nephrology [34]	content, citation, and sentiment analyses of tweets generated from Kidney Week 2011 would reveal a large number of educational tweets that were	Qualitative descriptive	5 days	Online observation	Deductive content analysis	moderate

Author/country/Social media type	Purpose	Design	Sample/data corpus	Data collection	Data analysis	Quality
	disseminated to the public.					
McGowan 2012<br>United states<br>Oncologist + primary care physicians<br>Social media[35]	assess factors related to social media use by physicians	Qualitative	Response rate 28.97% (485/1695)	Survey using Technology acceptance model<br>Cronbach α 0.92 (average)	Descriptive<br>Hierarchical regression	Strong
McKendrick 2012<br>USA<br>Anaesthetic clinicians<br>Twitter [36]	Describe the introduction and uptake of twitter at a conference	Qualitative descriptive	9 weeks	Online observation	Content analysis – deductive	Strong methods<br>Limited sample
Murty 2012<br>USA<br>Listserv<br>Social workers[37]	Categorise content of posts on mailing list	Qualitative descriptive	Data corpus 1 - 8 months<br>Data corpus 2 – 2 weeks<br>Data corpus 3 – 3 random months	Online observation	Content analysis - inductive	Strong
Stewart 2012<br>Thailand<br>Paediatric clinicians<br>Discussion forum [38]	To understand the dynamics of the knowledge sharing with the pediatric pain community	Social network analysis	27 months	Online observation	Descriptive & non-parametric<br>Social network analysis	
Usher 2012<br>Australia<br>Healthcare professionals<br>Social media[39]	Identifying the reason behind patterns of social media (Web 2.0) by 8 major healthcare professional groups	Survey	8 healthcare professional groups	Online survey<br>16 item instrument	Descriptive<br>Correlational	Fair
Von Muhlen 2012<br>Clinicians<br>Social media<br>[40]	Review social media adoption by clinicians	Literature review	Pubmed<br>To july 2011	Reviewed by primary author & scientific consultant; discrepancies resolved	Narrative<br>Summary table 1 only	Fair

Author/country/Social media type	Purpose	Design	Sample/data corpus	Data collection	Data analysis	Quality
				through discussion<br>Articles categorized into<br>1. Overviews<br>2. Adoption surveys<br>3. Reference use<br>4. Educational impact and use<br>5. Professional conduct		
Abrahamson 2013<br>International<br>Discussion forum<br>nurses[41]	To evaluate Information exchange in an online discussion forum ; identify potential for CoP	Qualitative descriptive	1 month discussion threads	Not described	Content analysis – deductive	Fair
Brynolf 2013<br>Sweden<br>Physicians<br>Twitter [42]	to investigate if unethical or unprofessional online behavior had occurred in a population based sample of Swedish speaking physicians and medical students on twitter	Qualitative descriptive	Swedish speaking medical officers<br>Last 100 tweets	Online observation	Content analysis – deductive	Moderate
Dieleman 2013<br>United Kingdom<br>Occupational therapist<br>Discussion forum [43]	Gain an understanding of the purpose and use of online discussion group	Case study	Data corpus – 8 years posts	Online observation<br>Census sampling	Theoretical Thematic analysis	Moderate
Hamm 2013	1. What social media	Literature review -	11 databases	Data extraction by	Narrative	Strong

Author/country/Social media type	Purpose	Design	Sample/data corpus	Data collection	Data analysis	Quality
Healthcare professionals<br>Social media in general<br>[44]	tools are being used by healthcare professionals and trainees?<br>2. In which disciplines and specialties are social media tools being used<br>3. For what purposes are social media tools being used<br>4. What types of evidence and research designs have been used to examine social media tools	scoping	2000-2012	single reviewer with 10% cross checked for accuracy<br>Data extracted<br>• Study & population characteristics<br>• Tool<br>• Objectives<br>• outcomes measure<br>• Authors' conclusions	Summary tables<br>1. Demographics of studies<br>2. Cross tabulation of tool type against objective of study<br>3. Setting against tool<br>4. Outcomes against tool	
Lulic 2013<br>USA<br>Emergency physicians<br>Twitter [45]	to identity and create the largest directory of emergency physicians on twitter; analyse their user profile and reveal details behind their connections	Exploratory descriptive	All twitter users self-identified as emergency physicians	Twiangulate, NodeXL, FollowWonk	descriptive	Fair
Moorhead 2013<br>Healthcare professionals social media[46]	to review the current published literature to identify the uses, benefits, and limitations of social media for health communication among the general public,	Literature review - systematic	10 databases<br>2002-2012	study design, social media tool/application, study purpose, participants/sample and sample size, measurement tools, results, conclusion, and use of social media	Summary tables<br>1. Social media tools/applications<br>2. Methodological qualities<br>3. By method<br>4. Uses of social media	Strong

Author/country/Social media type	Purpose	Design	Sample/data corpus	Data collection	Data analysis	Quality
	patients, and health professionals and to identify current gaps in the literature to provide recommendations for future health communication research.			two reviewers used Downs & Black	5. Benefits<br>6. Limitations	
Neill 2013<br>Twitter<br>Emergency medicine Conference tweeting [47]	To examine if twitter was a resource for disseminating clinical information and promoting and facilitating the aims of a medical conference	Mixed methods	All tweets with #ICEM2012 stratified	Online observation	Descriptive<br>Deductive content analysis	Strong
Anderson 2014<br>Australia<br>Public health<br>Twitter [48]	Explore what Twitter users communication and how they interacted across the conference days	Prospective descriptive	Census sample (3 days)	Online observation (Storify)	Thematic	satisfactory
Ferguson 2014<br>Australia and New Zealand<br>Cardiology clinicians<br>Twitter[49]	To evaluate twitter use during a national scientific meeting	Qualitative descriptive	Census	Online observation	Descriptive	Moderate
Frisch 2014<br>Canada<br>Nurses<br>VCoP [50]	To evaluate whether VCoP from the perspective of users	Mixed methods<br>Descriptive – use of website<br>2 Surveys – member satisfaction<br>Interviews – involvement in Action	Monthly website metrics from inception<br>Census sample for survey<br>Purposive sample for interviews	Online<br>Electronic survey	Descriptive for website and survey<br>Thematic for survey	Survey – fair<br>Interviews - moderate

Author/country/Social media type	Purpose	Design	Sample/data corpus	Data collection	Data analysis	Quality
		groups and perceptions of network's activities and successes in achieving goals				
Fuoco 2015<br>Social media<br>Canada<br>Urologists[51]	Understand attitudes and practices of urologists with respect to social media use in personal and professional lives	Qualitative	Census of active members of professional association	Online and paper survey	Descriptive with Fisher's exact test to compare across demographics or use settings	Fair
Hajar 2014<br>USA<br>Pharmacists<br>Twitter<br>[52]	To identify the number of pharmacists with twitter accounts, their usage characteristics and their professional networking patters	Qualitative descriptive	Census<br>30 Tweets from 1/3 of accounts	FollowerWonk	Tweets – inductive content analysis<br>SNA – NodeXL	Moderate
Hawkins 2014<br>International<br>Radiology professionals<br>Twitter [53]	To assess and quantify the use of twitter during a radiology conference	Descriptive	Census sample 20 days (Meeting six days + 1 week either side)	Online using Symplur[1]	Quantitative	Satisfactory
Kim 2014<br>Korea<br>Emergency physicians<br>Facebook [54]	Examine use of facebook page over initial 12 months	Mixed methods<br>Online observation<br>Survey	Census	Online observation of posts<br>Survey – paper, email telephone and facebook messaging	Posts – deductive content analysis<br>Survey - descriptive	Moderate
Matta 2014<br>North America<br>Physicians	To analyze the content of twitter activity for 2 national urology	Qualitative descriptive	Census sample covering conference period only	Online using Symplur[2]	Content analysis - deductive	Fair

[1] Symplur LLC Upland California USA
[2] Symplur California

Author/country/Social media type	Purpose	Design	Sample/data corpus	Data collection	Data analysis	Quality
Twitter[55]	meetings over two years					
Mishori 2014<br>USA<br>Physicians<br>Twitter[56]	Characterize and understand information diffusion in social media (Twitter) by examining twitter networks of 4 professional medical societies	Descriptive	Three months<br>Tweets – census sample – one month	Online observation<br>Topsy	Descriptive<br>Visualization	satisfactory
Mishori 2014<br>USA<br>Physicians<br>Twitter[57]	Analyze conference tweets to see who is talking and what they are talking about	Qualitative descriptive<br>- Tweet analysis<br>- Interviews	Census sample (8 days; 3 pre/post conference + 5 conference days<br>Top 9 tweeters	Online observation (Hootsuite and Hashtracking)<br>Email interviews	Deductive content analysis<br>thematic	Moderate (CA)<br>Limited for interviews
Moorley 2014<br>UK<br>Nurses<br>Twitter [58]	Evaluate the development, growth and positive experiences of using Twitter to create an online community including benefits, barriers and enablers	Qualitative descriptive	Census	Online observation	Descriptive	Fair
Rolls 2014<br>Australia<br>Intensive care<br>Listserv[59]	Describe the social network of a listserv for intensive care clinicians	Retrospective descriptive	Database<br>Census	Excel spreadsheet	Descriptive with some inferential	satisfactory
Ying Mai 2014<br>USA<br>Nurses<br>Social media[60]	Provide a preliminary review of the characteristics of nurses involved in social media use	Survey	160 professional advance practice nursing organizations and colleges of nursing	Survey, online	Descriptive with some inferential	Fair
Canvasser 2015<br>International	Examine use of Twitter by urologists by	Qualitative descriptive	Census sample 7 days (1 pre/post + 5	Online observation (Tweetreach)	Deductive content analysis (manual and	Moderate

Author/country/Social media type	Purpose	Design	Sample/data corpus	Data collection	Data analysis	Quality
Urologists<br>Twitter [61]	evaluating use during annual meeting		conference days)		machine (Semantria)	
Benetoli 2015<br>Pharmacists<br>Social media [62]	Review the literature on the social media use in professional pharmacy practice; and assess research designs used	Systematic literature review	Census			Adequate
Deen 2013<br>Mental health practitioners<br>US [63]	Identify to current use of social media and electronic communication by psychiatrists and psychologists, and their attitudes towards these platforms that hinder or facilitate care in the future	Survey; online	Census sample of academic faculty	Online	Descriptive with comparisons across groups; especially in respect to age	Limited
Klee 2015<br>Family medicine<br>USA<br>Social media [64]	Provide insight into family physicians' use and acceptance of social media; assess current professional training	Survey; online	Census sample of one state	Online	Descriptive with comparisons across group with respect to years of experience	Limited
Lawson 2015<br>Radiology<br>Australia<br>Social media [65]	What does the current literature report as common uses of social media for professional development in healthcare globally? How is social media used as professional development in	Systematic literature review	Academic literature post 2011		Descriptive	Limited

Author/country/Social media type	Purpose	Design	Sample/data corpus	Data collection	Data analysis	Quality
	healthcare?					
Grindrod 2014<br>Social media<br>International<br>Pharmacy [66]	Review how pharmacists and pharmacy students participate in social media and identify available guidance for professional behaviour	Scoping review; following framework	Medline, Embase, Google Scholar and International Pharmaceutical Abstracts for English articles published Pre May 2013		Thematic	fair
Tunnecliff 2015<br>Social media<br>Pacific [67]	To explore health researchers and clinicians current use of social media and their beliefs and attitudes towards the use of social media in professional context	Mixed methods<br>Online survey<br>Interviews	Targeted distribution to via research centres, department heads, professional organisations, affiliates of Monash university<br>Interviews self-nominated then randomly selected	Online researcher developed survey<br>Semi-structured telephone interviews	Descriptive and exploratory analysis of survey<br>Thematic analysis of qualitative data [68]	Survey – Fair<br>Interviews – moderate
Awad 2015<br>Pharmacy<br>Twitter<br>USA [69]	Evaluate the use of twitter by attendee and non-attendee participants in ASHPs 2013 and analyze the potential education utility	Qualitative descriptive	All tweets during conference	Symplur	Content analysis – deductive	Moderate
Loeb 2014<br>Physician - Urology<br>Social media<br>USA [70]	To characterize the current status of social media among AUA members and participation at 2013 meeting	Mixed methods<br>Survey<br>Online observation (#AUA13	Survey – random sample	Paper survey<br>Symplur	Descriptive	Fair
Whitaker 2003<br>Pharmacy<br>Listserv	1. Classify the topics discussed during one month	Mixed methods<br>Survey<br>Content analysis	Survey – census sample<br>One month of discussion threads	Online survey<br>Online observation	Survey – descriptive<br>Content analysis – deductive	Survey – moderate<br>Content

Author/country/Social media type	Purpose	Design	Sample/data corpus	Data collection	Data analysis	Quality
United Kingdom [71]	2. Survey the usage of and attitudes towards a mailing list for pharmacists<br>3. Identify the benefits of membership<br>4. Identify any changes in practice as a result of information from the list					analysis – fair
Roberts 2015<br>Healthcare<br>Twitter<br>International [72]	Evaluate status of social media facilitated journal clubs (twitter) as an example of continuing professional development	Systematic review + online observation	Medline, Embase, CINAHL, Web of Science, ERIC<br>Online search of Twitter	Online	Descriptive	Adequate
Barnett 2012<br>GP training<br>Virtual communities<br>International [73]	Critical review to<br>1. determine if there is any evidence to support virtual communities of practice in GP training;<br>2. Identify evidence-based guidelines for establishing VCoP	Literature review	Scopus, Psychlit and Pubmed		Thematic based on business virtual community framework [74]	Adequate

References

1. Berman Y. Discussion groups on the Internet as sources of information: the case of social work. Aslib Proceedings 1996;**48**(2):31-36. http://dx.doi.org/10.1108/eb051407.
2. Murray PJ. Nurses' computer-mediated communications on NURSENET: a case study. Computers in Nursing 1996;**14**(4):227-234. PMID: 8718843
3. Schoch NA, Shooshan SE. Communication on a listserv for health information professionals: uses and users of MEDLIB-L. Bulletin of the Medical Library Association 1997;**85**(1):23-32. PMID:PMC226219
4. Roberts C, Fox N. General practitioners and the Internet: modelling a 'virtual community'. Family Practice 1998;**15**(3):211-15. PMID: 9694177
5. Murray PJ. Subject: talk.to/reflect - reflection and practice in nurses' computer-mediated communications, in Institute of Educational Technology. 2001, The Open University. p. 338 ^http://www.nursing-informatics.com/PhD/PMurray.pdfJanuary 8 2008.
6. Reutzel TJ, Patel R. Medication management problems reported by subscribers to a school nurse listserv. The Journal of School Nursing 2001;**17**(3):131-13910.1177/10598405010170030401. PMID:11885443
7. Cervantez Thompson TL. You've got mail: Rehabilitation nurses on the RehabNurse-L LISTSERV. Rehabilitation Nursing 2002;**27**(4):146-151. PMID:12116527
8. Watson DAR. Ozbug: an email mailing list for physicians that works. Internal Medicine Journal 2003;**33**(11):532-534. PMID:14656258
9. Cervantez Thompson TLPenprase B. RehabNurse-L: An Analysis of the Rehabilitation Nursing LISTSERV Experience. Rehabilitation Nursing 2004;**29**(2):56-6111/2/2008).10.1002/j.2048-7940.2004.tb00307.x. PMID:15052747
10. Brooks F, Scott P. Exploring knowledge work and leadership in online midwifery communication. Journal of Advanced Nursing 2006;**55**(4):510-20. PMID:16866846
11. Brooks F, Scott P. Knowledge work in nursing and midwifery: an evaluation through computer-mediated communication. International Journal of Nursing Studies 2006;**43**:83-97. PMID:16326164
12. Hara N, Hew K. Knowledge-sharing in an online community of health care professionals. Information, Technology and People 2007;**20**(3):235-261. DOI:10.1108/09593840710822859.
13. Hew KF, Hara N. Knowledge sharing in online environments: a qualitative case study. Journal of the American Society for Information Science and Technology 2007;**58**(14):2310-2324. DOI: 10.1002/asi.20698.
14. Rodriguez-Recio FJ, Sendra-Portero F. Analysis of the Spanish-speaking mailing list RADIOLOGIA. European Journal of Radiology 2007;**63**:136-143. PMID: 17344009
15. Hew KF, Hara N. An online listserv for nurse practitioners: a viable venue for continuous nursing professional development? Nurse Education Today 2008;**28**(4):450-71. PMID:17881096
16. Rolls K, Kowal D, Elliott D, Burrell A. Building a statewide knowledge network for clinicians in intensive care units: knowledge brokering and the NSW Intensive Care Coordination and Monitoring Unit. Australian Critical Care 2008;**21**(1):27-37. PMID:18226542
17. Widemark E. Community and Learning: A Virtual Community of Practice for Nurse Practitioners, in Education. 2008, Capella University: Ann Arbour, United States, 8 June 2009.

18. Cook-Craig PG, Sabah Y. The role of virtual communities of practice in supporting collaborative learning among social workers. British Journal of Social Work 2009;**39**:725-739. DOI:10.1093/bjsw/bcp048.
19. Hughes B, Joshhi I, Lemonde H, Wareham J. Junior physician's use of Web 2.0 for information seeking and medical education. International Journal of Medical Informatics 2009;**78**:645-655. PMID:19501017
20. Long S, de Jonge D, Ziviani J, Jones A. Paediatricots: utilisation of an Australian list serve to support occupational therapists working with children. Australian Occupational Therapy Journal 2009;**56**(1):63-71. PMID:20854490
21. Macdonald L, MacPherson DW, Gushulak BD. Online communication as a potential travel medicine research tool: analysis of messages posted on the TravelMed listserv. Journal of Travel Medicine 2009;**16**(1):7-12. PMID:19192121
22. Morken T, Bull N, Moen BE. The activity on a Norwegian Occupational Health mailing list 1997-2006. Occupational Medicine 2009;**59**:56-58. PMID:19001070
23. Shanahan M, Herrington A, Herrington J. The Internet and the medical radiation science practitioner. Radiography 2009;**15**(3):233-241. PMID: 25739107
24. Foong DP, McGrouther DA. An Internet-based discussion forum as a useful resource for the discussion of clinical cases and an educational tool. Indian journal of plastic surgery: official publication of the Association of Plastic Surgeons of India 2010;**43**(2):195. PMID:PMC3010782
25. Franko OI. Twitter as a communication tool for orthopedic surgery. Orthopedics 2011;**34**(11):873-876. PMID: 22050252.
26. Hoffmann T, Desha L, Verrall K. Evaluating an online occupational therapy community of practice and its role in supporting occupational therapy practice. Australian Journal of Occupational Therapy 2011;**58**:337-345. PMID:21957918
27. Kukreja P, Heck Sheehan A, Riggins J. Use of social media by pharmacy preceptors. Am J Pharm Educ 2011;**75**(9):176. PMID:PMC3230337
28. Lau ASM. Hospital-Based Nurses' Perceptions of the Adoption of Web 2.0 Tools for Knowledge Sharing, Learning, Social Interaction and the Production of Collective Intelligence. Journal of Medical and Internet Research 2011;**13**(4):e92. PMID:22079851
29. Valaitis RK, Akhtar-Danesh N, Brooks F, Vings S, Semogas D. Online communities of practice as a communication resource for community health nurses working with homeless people. Journal of Advanced Nursing 2011;**67**(6):1273-1284. PMID:21306424
30. Apostolakis I, Koulierakis G, Berler A, Chryssanthou A, Varlamis I. Use of social media by healthcare professionals in Greece: an exploratory study. International Journal of Electronic Healthcare 2012;**7**(2):105-24. PMID:23079026
31. Archambault PM, Bilodeau A, Gagnon M-P, Aubin K, Lavoie A, Lapointe J et al. Health Care Professionals' Beliefs About Using Wiki-Based Reminders to Promote Best Practices in Trauma Care. Journal of Medical and Internet Research 2012;**14**(2). PMID:22515985
32. Burg MA, Adorno G, Hidalgo J. An Analysis of Social Work Oncology Network Listserv Postings on the Commission of Cancer's Distress Screening Guidelines. Journal of psychosocial oncology 2012;**30**(6):636-651.PMID:23101548
33. Chaudhry A, Glodé LM, Gillman M, Miller RS. Trends in twitter use by physicians at the American Society of Clinical Oncology annual meeting, 2010 and 2011. Journal of Oncology Practice 2012;**8**(3):173-178. PMID: PMC3396806
34. Desai T, Shariff A, Shariff A, Kats M, Fang X, Christiano CFerris. Tweeting the meeting: an in-depth analysis of Twitter activity at Kidney Week 2011. PloS one 2012;**7**(7):e40253. PMID:PMC3390326

35. McGowan BS Wasko M, Vartabedian BS, Miller RS, Freiherr DD, Abdolrasulnia M.. Understanding the factors that influence the adoption and meaningful use of social media by physicians to share medical information. Journal of Medical and Internet Research 2012;**14**(5) 29/9/2012). PMID:23006336
36. McKendrick DRA, Cumming GP, Lee AJ. Increased use of Twitter at a medical conference: a report and a review of the educational opportunities. Journal of Medical and Internet Research 2012;**14**(6):e176.11 December 2012,(10.2196/jmir.2144. PMID:PMC3799570
37. Murty SA. Using a LISTSERV a community of practice in end-of-life, hospice, and palliative care social work. Journal of Social Work in End-of-Life & Palliative Care 2012;**8**(1):77-101 PMID:22424385
38. Stewart SA, Abidi SSR. Applying social network analysis to understand the knowledge sharing behaviour of practitioners in a clinical online discussion forum. Journal of medical Internet research 2012;**14**(6):e17029. PMID:PMC3799555
39. Usher WT. Australian health professionals' social media (Web 2.0) adoption trends: early 21st century health care delivery and practice promotion. Australian Journal of Primary Health 2012;**18**:31-41. PMID:22394660
40. von Muhlen MOhno-Machado L. Reviewing social media use by clinicians. Journal of the American Medical Informatics Association 2012;**19**(5):777-781.PMID:22759618
41. Abrahamson K, Fox R, Anderson JG. What nurses are talking about: content and community within a nursing online forum. Stud Health Technol Inform 2013;**183**:350-5.DOI: 10.3233/978-1-61499-203-5-350
42. Brynolf A, Johansson S, Appelgren E, Lynoe N, Edstedt Bonamy AK. Virtual colleagues, virtually colleagues--physicians' use of Twitter: a population-based observational study. BMJ Open 2013;**3**(7). PMID:PMC3731708
43. Dieleman C, Duncan EA. Investigating the purpose of an online discussion group for health professionals: a case example from forensic occupational therapy. BMC health services research 2013;**13**(1):25310. PMID:PMC3702402
44. Hamm MP, Chisholm A, Shulhan J, Milne A, Scott SD, Klassen TP, Hartling L. Social Media Use by Health Care Professionals and Trainees: A Scoping Review. Academic Medicine 2013;**88**(13):1376-1383. PMID:23887004
45. Lulic I, Kovic I. Analysis of emergency physicians' Twitter accounts. Emergency Medicine Journal 2013;**30**:371-376. PMID: 22634832
46. Moorhead SA Hazlett DE, Harrison L, Carroll JK, Irwin AHoving C. A new dimension of health care: systematic review of the uses, benefits, and limitations of social media for health communication. Journal of Medical and Internet Research 2013;**15**(4):e85. PMID:PMC3636326
47. Neill A Cronin JJ, Brannigan D, O'Sullivan R, Cadogan M. The impact of social media on a major international emergency medicine conference. Emergency Medicine Journal 2014;**31**(5):401-4. PMID:23423992
48. Anderson G, Gleeson S, Rissel C, Wen LMBedford K. Twitter tweets and twaddle: twittering at AHPA. Health Promotion Journal of Australia 2014;**25**(2):143-146. PMID:25200470
49. Ferguson C, Inglis SC, Newton PJ, Cripps PJS. Social media: A tool to spread information: A case study analysis of Twitter conversation at the Cardiac Society of Australia and New Zealand 61st Annual Scientific Meeting 2013. Collegian 2014;**21**(2):89-93. PMID:25109206
50. Frisch N, Atherton P, Borycki E, Mickelson G, Cordeiro J, Novak Lauscher H, Black A. Growing a Professional Network to Over 3000 Members in Less Than 4 Years: Evaluation of InspireNet, British Columbia's Virtual Nursing Health Services Research Network. Journal of Medical and Internet Research 2014;**16**(2):e4910. PMID:24566806

51. Fuoco M, Leveridge MJ. Early adopters or laggards? Attitudes toward and use of social media among urologists. BJU International 2015;**115**(3):491-497. PMID:24981237
52. Hajar Z, Clauson KA, Jacobs RJ. Analysis of pharmacists' use of Twitter. American Journal of Health-System Pharmacy 2014;**71**(8):615-619. PMID:24688034
53. Hawkins CM, Duszak R, Rawson JV. Social Media in Radiology: Early Trends in Twitter Microblogging at Radiology's Largest International Meeting. Journal of the American College of Radiology 2014;**11**(4):387-390. PMID:24139963
54. Kim C. Nationwide online social networking for cardiovascular care in Korea using Facebook. J Am Med Inform Assoc 2014;**21**(1):17-22. PMID:PMC3912716
55. Matta R, Doiron C, Leveridge MJ. The Dramatic Increase in Social Media in Urology. The Journal of Urology 2014;**192**(2):494-498. PMID:24576656
56. Mishori R. Mapping Physician Twitter Networks: Describing How They Work as a First Step in Understanding Connectivity, Information Flow, and Message Diffusion. J Med Internet Res 2014;**16**(4):e107.14.04.2014,(22 October 2014).10.2196/jmir.3006. PMID:24733146
57. Mishori R, Levy B, Donvan B. Twitter Use at a Family Medicine Conference: Analyzing# STFM13. Family medicine 2014;**46**(8):608-614. PMID:25163039
58. Moorley CR, Chinn T. Nursing and Twitter: Creating an online community using hashtags. Collegian 2014;**21**(2):103-109. PMID: 25109208
59. Rolls K, Hansen M, Jackson D, Elliott D. Analysis of the Social Network Development of a Virtual Community for Australian Intensive Care Professionals. Computers Informatics Nursing 2014;**32**(11):536-544. PMID:25310223
60. Ying Mai K, Sanghee O. Characteristics of Nurses Who Use Social Media. CIN: Computers, Informatics, Nursing 2014;**32**(2):64-72. PMID: 24419089
61. Canvasser NE, Ramo C, Morgan TM, Zheng K, Hollenbeck BK, Ghani KR. The use of social media in endourology: an analysis of the 2013 World Congress of Endourology meeting. Journal of Endourology 2015;**29**(5):615-620. PMID:25026076
62. Benetoli A, Chen TF, Aslani P. The use of social media in pharmacy practice and education. Research in Social and Administrative Pharmacy 2015;**11**(1):1-46. PMID:24814268
63. Deen SR, Withers A, Hellerstein DJ. Mental Health Practitioners' Use and Attitudes Regarding the Internet and Social Media. Journal of Psychiatric Practice® 2013;**19**(6):454-463. PMID:24241499
64. Klee D, Covey C, Zhong L. Social media beliefs and usage among family medicine residents and practicing family physicians. Family medicine 2015;**47**(3):222-226. PMID:25853534
65. Lawson C, Cowling C. Social media: The next frontier for professional development in radiography. Radiography 2015;**21**(2):e74-e80. http://dx.doi.org/10.1016/j.radi.2014.11.006.
66. Grindrod K, Forgione A, Tsuyuki RT, Gavura S, Giustini D. Pharmacy 2.0: a scoping review of social media use in pharmacy. Research in Social and Administrative Pharmacy 2014;**10**(1):256-270. PMID:23810653
67. Tunnecliff J, Ilic D, Morgan P, Keating J, Gaida JE, Clearihan L, etal. The Acceptability Among Health Researchers and Clinicians of Social Media to Translate Research Evidence to Clinical Practice: Mixed-Methods Survey and Interview Study. Journal of Medical Internet Research 2015;**17**(5):e11910.2196/jmir.4347. PMID:4468567

68. Braun V, Clarke V. Using thematic analysis in psychology. Qualitative research in psychology 2006;**3**(2):77-101. DOI:10.1191/1478088706qp063oa.
69. Awad NI, Cocchio C. Use of Twitter at a major national pharmacy conference. American Journal of Health-System Pharmacy 2015;**72**(1):65-69. PMID:25511841
70. Loeb S, Bayne CE, Frey C, Davies BJ, Averch TD, Woo HH etal. Use of social media in urology: data from the American Urological Association (AUA). BJU international 2014;**113**(6):993-998. PMID:24274744
71. Whitaker S, Cox AR, Alexander AM. Internet networking for pharmacists: an evaluation of a mailing list for UK pharmacists. International Journal of Pharmacy Practice 2003;**11**(1):25-32. DOI:10.1211/002235702784.
72. Roberts MJ, Perera M, Lawrentschuk N, Romanic D, Papa NBolton D. Globalization of Continuing Professional Development by Journal Clubs via Microblogging: A Systematic Review. Journal of medical Internet research 2015;**17**(4). PMID:4424319
73. Barnett S, Jones S, Bennett S, Iverson DBonney A. General practice training and virtual communities of practice-a review of the literature. BMC family practice 2012;**13**(1):8710.1186/1471-2296-13-87. PMID:22905827
74. Probst B, Borzillo S. Why communities of practice succeed and why they fail. European Journal of Management 2008;**26**:335-347.
