# Supplementary material for: How Health Care Professionals Use Social Media to Create Virtual Communities: An Integrative Review
Source: J Med Internet Res. 2016 Jun 16;18(6):e166. doi: 10.2196/jmir.5312 (PMC4933801; doi:10.2196/jmir.5312)
Supplement: Multimedia Appendix 3 [file jmir_v18i6e166_app3.pdf]

CASP [1]criteria

1. Was there a clear statement of the aims of the research?
2. Is a qualitative methodology appropriate?
3. Was the research design appropriate to address the aims of the research?
4. Was the recruitment strategy appropriate to the aims of the research?
5. Was the data collected in a way that addressed the research issue?
6. Has the relationship between researcher and participants been adequately considered?
7. Have ethical issues been taken into consideration?
8. Was the data analysis sufficiently rigorous?
9. Is there a clear statement of findings?
10. How valuable is the research?

Author	1	2	3	4	5	6	7	8	9	10	quality
Murray 1996 [2]	yes	yes	yes	yes	yes	cant tell	yes	cant tell	yes	yes	satisfactory
Roberts 1998 [3]	yes	yes	yes	yes	yes	yes	cant tell	yes	yes	yes	satisfactory
Cervantez-thompson 2004 [4]	yes	yes	yes	yes	yes	cant tell	yes	cant tell	yes	yes	satisfactory
Brooks 2006 [5]	yes	yes	yes	yes	yes	cant tell	yes	cant tell	yes	yes	satisfactory
Hara 2007 [6]	yes	yes	yes	cant tell	yes	cant tell	yes	yes	yes	yes	satisfactory
Hew 2007 [7]	yes	yes	yes	yes	yes	cant tell	yes	yes	yes	yes	satisfactory
Hew 2008 [8]	yes	yes	yes	yes	yes	yes	yes	yes	yes	yes	satisfactory
Hughes 2009 [9]	yes	yes	yes	yes	yes	cant tell	cant tell	yes	yes	yes	satisfactory
Valaitis 2011 [10]	yes	yes	yes	yes	yes	cant tell	yes	yes	yes	yes	satisfactory
Archamabault 2012[11]	yes	yes	yes	yes	yes	cant tell	yes	yes	yes	yes	satisfactory
Dieleman 2013 [12]	yes	yes	yes	cant tell	cant tell	No	cant tell	yes	yes	yes	satisfactory
Anderson 2014 [13]	yes	yes	yes	yes	yes	cant tell	cant tell	yes	yes	cant tell	satisfactory
Ferguson 2014 [14]	yes	cant tell	cant tell	yes	yes	yes	yes	yes	yes	yes	satisfactory
Frisch 2014 [15]	yes	cant tell	yes	yes	yes	yes	cant tell	yes	cant tell	yes	satisfactory
Moorley 2014 [16]	yes	yes	not sure	cant tell	yes	no	no	no	no	yes	Satisfactory
Tunnecliff 2015 [17]	yes	yes	yes	yes	yes	no	cant tell	cant tell	cant tell	yes	satisfactory

1. International C. Qualitative Research Checklist, in Critical Appraisal Tools. 2013, CASP International: United Kingdom^http://www.caspinternational.org/mod_product/uploads/CASP%20Qualitative%20Research%20Checklist%2031.05.13.pdf4 February 2014.
2. Murray PJ. Nurses' computer-mediated communications on NURSENET: a case study. Computers in Nursing 1996;**14**(4):227-234. PMID: 8718843
3. Roberts C, Fox N. General practitioners and the Internet: modelling a 'virtual community'. Family Practice 1998;**15**(3):211-156/2/2008).10.1093/fampra/15.3.211. PMID: 9694177
4. Cervantez Thompson TL, Penprase B. RehabNurse-L: An Analysis of the Rehabilitation Nursing LISTSERV Experience. Rehabilitation Nursing 2004;**29**(2):56-61. PMID:15052747
5. Brooks FScott P. Exploring knowledge work and leadership in online midwifery communication. Journal of Advanced Nursing 2006;**55**(4):510-20. PMID:16866846
6. Hara N, Hew K. Knowledge-sharing in an online community of health care professionals. Information, Technology and People 2007;**20**(3):235-261. DOI:10.1108/09593840710822859.
7. Hew K, FHara N. Knowledge sharing in online environments: a qualitative case study. Journal of the American Society for Information Science and Technology 2007;**58**(14):2310-2324. DOI:10.1002/asi.20698.
8. Hew KF, Hara N. An online listserv for nurse practitioners: a viable venue for continuous nursing professional development? Nurse Education Today 2008;**28**(4):450-71. PMID:17881096
9. Hughes B, Joshi I, Lemonde H, Wareham J. Junior physician's use of Web 2.0 for information seeking and medical education. International Journal of Medical Informatics 2009;**78**:645-655. PMID:19501017
10. Valaitis RK, Akhtar-Danesh N, Brooks F, Vings S, Semogas D. Online communities of practice as a communication resource for community health nurses working with homeless people. Journal of Advanced Nursing 2011;**67**(6):1273-1284. PMID:21306424
11. Archambault PM, Bilodeau A, Gagnon M-P, Aubin K, Lavoie A, Lapointe J et al. Health Care Professionals' Beliefs About Using Wiki-Based Reminders to Promote Best Practices in Trauma Care. Journal of Medical and Internet Research 2012;**14**(2). PMID:22515985
12. Dieleman C, Duncan EA. Investigating the purpose of an online discussion group for health professionals: a case example from forensic occupational therapy. BMC health services research 2013;**13**(1):25310. PMID:PMC3702402
13. Anderson G, Gleeson S, Rissel C, Wen LM, Bedford K. Twitter tweets and twaddle: twittering at AHPA. Health Promotion Journal of Australia 2014;**25**(2):143-146. PMID:25200470
14. Ferguson C, Inglis SC, Newton PJ, Cripps PJS, Macdonald PS, Davidson PM. Social media: A tool to spread information: A case study analysis of Twitter conversation at the Cardiac Society of Australia and New Zealand 61st Annual Scientific Meeting 2013. Collegian 2014;**21**(2):89-93. PMID:25109206
15. Frisch N, Atherton P, Borycki E, Mickelson G, Cordeiro J, Novak Lauscher H, Black A. Growing a Professional Network to Over 3000 Members in Less Than 4 Years: Evaluation of InspireNet, British Columbia's Virtual Nursing Health Services Research Network. Journal of Medical and Internet Research 2014;**16**(2):e49. PMID:24566806
16. Moorley CR, Chinn T. Nursing and Twitter: Creating an online community using hashtags. Collegian 2014;**21**(2):103-109. PMID:25109208
17. Tunnecliff J, Ilic D, Morgan P, Keating J, Gaida JE, Clearihan L, et al. The Acceptability Among Health Researchers and Clinicians of Social Media to Translate Research Evidence to Clinical Practice: Mixed-Methods Survey and Interview Study. Journal of Medical Internet Research 2015;**17**(5):e119. PMID:4468567
