# Supplementary material for: How Health Care Professionals Use Social Media to Create Virtual Communities: An Integrative Review
Source: J Med Internet Res. 2016 Jun 16;18(6):e166. doi: 10.2196/jmir.5312 (PMC4933801; doi:10.2196/jmir.5312)
Supplement: Multimedia Appendix 4 [file jmir_v18i6e166_app4.pdf]

Quality assessment of studies using content analysis techniques [1-3] included:

- Data: appropriateness to research question, data corpus, sampling unit, unit of analysis and sampling plan (described and justified)
- Coding schema: appropriateness of approach, development, coders, training, theoretical underpinning of categories and reliability of coding schema
- Analysis: appropriateness of approach

References	Approach	RQ	Data corpus	Sampling unit	Unit of analysis	Sampling plan	Appropriate approach	Development	Coders	Training	Theoretical underpinning of categories	Reliability	Appropriateness of approach	Overall score
Berman 1996 [4]	deductive	✓	✓	×	×	✓	✓	×	×	×	×	×	×	✓
Bowers 1997 [5]	Inductive	✓	✓✓	✓✓	✓	✓	✓	✓	✓	×	✓	×	✓	✓
Cervantez Thompson 2002 [6]	deductive	✓	✓✓	✓✓	✓✓	✓✓	✓✓	✓	✓	×	✓	×	✓	✓
Whitaker 2003 [7]	deductive	✓✓	✓	✓	✓	✓	✓	✓✓	✓	✓	✓	✓	✓	✓
Smith 2004[8]	deductive	✓	✓✓	✓✓	✓	✓✓	✓	✓	×	×	✓	×	✓	✓
(Rodriguez-Recio 2007) [9]	deductive	✓✓	✓✓✓	✓	✓	✓✓✓	✓✓	✓	×	×	✓	×	×	✓

References	Approach	RQ	Data corpus	Sampling unit	Unit of analysis	Sampling plan	Appropriate approach	Development	Coders	Training	Theoretical underpinning of categories	Reliability	Appropriateness of approach	Overall score
Morken 2009 [10]	Inductive	✓	✓	✓	✓	✓✓	✓	✓	✓	✓	✓	✓✓	✓	✓
Foong 2010[11]	deductive	✓✓	✓✓✓	✓✓	✓✓	✓✓	✓✓	×	×	×	✓	×	×	✓
Abrahamson 2013 [12]	Inductive	✓	✓	✓✓	✓✓	✓	✓	✓	✓✓	✓✓	✓	✓	✓	✓
Matta 2014 [13]	deductive	✓	✓✓	✓✓	✓✓	✓✓	✓✓	×	×	×	✓	×	×	✓
Murray 1996 [14]	Inductive	✓✓✓	✓✓✓	✓✓✓	✓✓✓	✓✓✓	✓✓✓	✓✓	✓	✓	✓	✓	✓✓	✓✓
Long 2009 [15]	Inductive	✓✓	✓✓✓	✓✓	✓	✓✓	✓✓	✓✓✓	✓✓	✓	✓✓✓	✓✓	✓✓	✓✓
Macdonald 2009 [16]	deductive	✓✓	✓✓	✓✓	✓	✓✓	✓✓	✓✓	✓	✓	✓✓	✓	✓	✓✓
Chaudhry 2012 [17]	deductive	✓✓	✓✓✓	✓✓✓	✓✓✓	✓✓✓	✓✓✓	✓✓	✓✓	✓✓	✓✓	✓	✓✓	✓✓
Hajar 2014 [18]	inductive	✓✓	✓✓	✓✓	✓✓✓	✓✓	✓✓	✓✓✓	✓✓	×	✓✓	✓✓✓	✓✓	✓✓

References	Approach	RQ	Data corpus	Sampling unit	Unit of analysis	Sampling plan	Appropriate approach	Development	Coders	Training	Theoretical underpinning of categories	Reliability	Appropriateness of approach	Overall score
Canvasser 2015 [19]	deductive	✓	✓✓✓	✓✓	✓✓	✓✓✓	✓✓✓	✓✓✓	✓✓✓	✓✓✓	✓✓	✓✓	✓✓	✓✓
Awad 2015 [20]	deductive	✓✓	✓✓	✓✓	✓✓	✓✓	✓✓	✓✓	✓	✓	✓✓	✓✓	✓✓	✓✓
Mishori 2014 [21]	deductive	✓✓	✓✓✓	✓✓	✓✓✓	✓✓	✓✓	✓✓	✓✓✓	✓✓	✓	✓✓	✓✓	✓✓
Reutzel 2001[22]	deductive	✓✓✓	✓✓✓	✓✓	✓✓✓	✓✓✓	✓✓✓	✓✓✓	✓✓	✓✓	✓✓✓	✓✓	✓✓✓	✓✓✓
Brooks 2006a [23]	deductive	✓✓✓	✓✓	✓✓	✓✓	✓✓	✓✓	✓✓	✓✓✓	✓✓✓	✓✓✓	✓✓✓	✓✓✓	✓✓✓
Brooks 2006b[24]	deductive	✓✓✓	✓✓	✓✓	✓✓	✓✓✓	✓✓	✓✓✓	✓✓✓	✓✓✓	✓✓✓	✓✓	✓✓✓	✓✓✓
Hara 2007[25]	Inductive	✓✓✓	✓✓✓	✓✓✓	✓✓✓	✓✓✓	✓✓✓	✓✓✓	✓✓✓	✓✓✓	✓✓	✓✓✓	✓✓✓	✓✓✓
Hew 2007 [26]	Inductive	✓✓✓	✓✓✓	✓✓✓	✓✓✓	✓✓✓	✓✓✓	✓✓✓	✓✓✓	✓✓✓	✓✓✓	✓✓✓	✓✓✓	✓✓✓
Burg2012 [27]	Inductive	✓✓✓	✓✓✓	✓✓✓	✓✓	✓✓✓	✓✓✓	✓✓✓	✓✓✓	✓✓✓	✓✓	✓✓✓	✓✓✓	✓✓✓

References	Approach	RQ	Data corpus	Sampling unit	Unit of analysis	Sampling plan	Appropriate approach	Development	Coders	Training	Theoretical underpinning of categories	Reliability	Appropriateness of approach	Overall score
Desai 2012[28]	deductive	✓✓✓	✓✓✓	✓✓	✓✓✓	✓✓✓	✓✓	✓✓✓	✓✓	✓✓	✓✓✓	✓	✓✓	✓✓✓
McKendrick 2012) [29]	deductive	✓✓	✓✓	✓✓	✓✓	✓✓	✓✓	✓✓✓	✓✓✓	✓✓✓	✓✓✓	✓✓✓	✓✓✓	✓✓✓
Murty 2012 [30]	Inductive	✓✓	✓✓✓	✓✓✓	✓	✓✓✓	✓✓✓	✓✓✓	✓✓✓	✓✓✓	✓✓✓	✓✓	✓✓✓	✓✓✓
Neill 2014[31]	deductive	✓✓✓	✓✓✓	✓✓✓	✓✓✓	✓✓✓	✓✓✓	✓✓✓	✓✓	✓✓	✓✓	✓✓	✓✓✓	✓✓✓
Brynolf 2013 [32]	deductive	✓✓✓	✓✓✓	✓✓✓	✓✓✓	✓✓✓	✓✓✓	✓✓	✓	✓✓	✓✓✓	✓	✓✓✓	✓✓✓
Kim [33]	deductive	✓✓	✓✓✓	✓✓✓	✓✓✓	✓✓✓	✓✓✓	✓✓	✓✓✓	✓✓	✓✓	✓✓✓	✓✓✓	✓✓✓

References

1. Krippendorff K. Content analysis: an introduction to its methodology. 2nd ed. Thousand Oaks, California, USA.: SAGE; 2004. ISBN:978-0-7619-1545-4
2. Zhang Y, Wildemuth BM. Qualitative analysis of content, In B.M. Wildemuth, editors.Applications of social research methods to questions in information and library science. Westport. Libraries Unlimited.2009 p. 308-319.
3. Graneheim UH, Lundman B. Qualitative content analysis in nursing research: concepts, procedures and measures to achieve trustworthiness. Nurse education today 2004;**24**(2):105-112. PMID:14769454

4. Berman Y. Discussion groups on the Internet as sources of information: the case of social work. Aslib Proceedings 1996;**48**(2):31-36. DOI:http://dx.doi.org/10.1108/eb051407.
5. Bowers L. Constructing international professional identity: what psychiatric nurses talk about on the Internet. International Journal of Nursing Studies 1997;**34**(3):208-212. PMID: 9219053
6. Cervantez Thompson TL. You've got mail: Rehabilitation nurses on the RehabNurse-L LISTSERV. Rehabilitation Nursing 2002;**27**(4):146-151. PMID:12116527
7. Whitaker S, Cox AR, Alexander AM. Internet networking for pharmacists: an evaluation of a mailing list for UK pharmacists. International Journal of Pharmacy Practice 2003;**11**(1):25-32. DOI:10.1211/002235702784.
8. Smith C. A longitudinal study of the culture of MEDLIB-L. Journal of Hospital Librarianship 2004;**4**(1):29-42. PMID:14656258
9. Rodriguez-Recio FJSendra-Portero F. Analysis of the Spanish-speaking mailing list RADIOLOGIA. European Journal of Radiology 2007;**63**:136-143. PMID: 17344009
10. Morken T, Bull N, Moen BE. The activity on a Norwegian Occupational Health mailing list 1997-2006. Occupational Medicine 2009;**59**:56-58. PMID:19001070
11. Foong DP, McGrouther DA. An Internet-based discussion forum as a useful resource for the discussion of clinical cases and an educational tool. Indian journal of plastic surgery 2010;**43**(2):19510. PMID:PMC3010782
12. Abrahamson K, Fox R, Anderson JG. What nurses are talking about: content and community within a nursing online forum. Stud Health Technol Inform 2013;**183**:350-5.DOI:10.3233/978-1-61499-203-5-350.
13. Matta R, Doiron C, Leveridge MJ. The Dramatic Increase in Social Media in Urology. The Journal of Urology 2014;**192**(2):494-498. PMID:24576656
14. Murray PJ. Nurses' computer-mediated communications on NURSENET: a case study. Computers in Nursing 1996;**14**(4):227-234. PMID: 8718843
15. Long S, de Jonge D, Ziviani J, Jones A. Paediatricots: utilisation of an Australian list serve to support occupational therapists working with children. Australian Occupational Therapy Journal 2009;**56**(1):63-71. PMID:20854490
16. Macdonald L, MacPherson DW, Gushulak BD. Online communication as a potential travel medicine research tool: analysis of messages posted on the TravelMed listserv. Journal of Travel Medicine 2009;**16**(1):7-12. PMID:19192121
17. Chaudhry A, Glodé LM, Gillman M, Miller RS. Trends in twitter use by physicians at the American Society of Clinical Oncology annual meeting, 2010 and 2011. Journal of Oncology Practice 2012;**8**(3):173-178. PMID: PMC3396806
18. Hajar Z, Clauson KAJacobs RJ. Analysis of pharmacists' use of Twitter. American Journal of Health-System Pharmacy 2014;**71**(8):615-619 PMID:24688034
19. Canvasser NE, Ramo C, Morgan TM, Zheng K, Hollenbeck BK, Ghani KR. The use of social media in endourology: an analysis of the 2013 World Congress of Endourology meeting. Journal of Endourology 2015;**29**(5):615-620. PMID:25026076
20. Awad NICocchio C. Use of Twitter at a major national pharmacy conference. American Journal of Health-System Pharmacy 2015;**72**(1):65-69 PMID:25511841
21. Mishori R, Levy B, Donvan B. Twitter Use at a Family Medicine Conference: Analyzing# STFM13. Family medicine 2014;**46**(8):608-614 PMID:25163039
22. Reutzel TJ, Patel R. Medication management problems reported by subscribers to a school nurse listserv. The Journal of School Nursing 2001;**17**(3):131-139. PMID:11885443
23. Brooks F, Scott P. Knowledge work in nursing and midwifery: an evaluation through computer-mediated communication. International Journal of Nursing Studies 2006;**43**:83-97. PMID:16326164

24. Brooks F, Scott P. Exploring knowledge work and leadership in online midwifery communication. Journal of Advanced Nursing 2006;**55**(4):510-20 PMID:16866846
25. Hara N, Hew K. Knowledge-sharing in an online community of health care professionals. Information, Technology and People 2007;**20**(3):235-261 DOI:10.1108/09593840710822859.
26. Hew KF, Hara N. Knowledge sharing in online environments: a qualitative case study. Journal of the American Society for Information Science and Technology 2007;**58**(14):2310-2324. DOI:10.1002/asi.20698.
27. Burg MA, Adorno G, Hidalgo J. An Analysis of Social Work Oncology Network Listserv Postings on the Commission of Cancer's Distress Screening Guidelines. Journal of psychosocial oncology 2012;**30**(6):636-651. PMID:23101548
28. Desai T, Shariff A, Shariff A, Kats M, Fang X, Christiano C, Ferris M. Tweeting the meeting: an in-depth analysis of Twitter activity at Kidney Week 2011. PloS one 2012;**7**(7):e40253. PMID:PMC3390326
29. McKendrick DRA, Cumming GP, Lee AJ. Increased use of Twitter at a medical conference: a report and a review of the educational opportunities. Journal of Medical and Internet Research 2012;**14**(6):e176. PMID:PMC3799570
30. Murty SA, Gilmore K, Richards KA, Altilio T. Using a LISTSERV a community of practice in end-of-life, hospice, and palliative care social work. Journal of Social Work in End-of-Life & Palliative Care 2012;**8**(1):77-101. PMID:22424385
31. Neill A, Cronin JJ, Brannigan D, O'Sullivan R, Cadogan M. The impact of social media on a major international emergency medicine conference. Emergency Medicine Journal 2014;**31**(5):401-4. PMID:23423992
32. Brynolf A, Johansson S, Appelgren E, Lynoe N, Edstedt Bonamy AK. Virtual colleagues, virtually colleagues--physicians' use of Twitter: a population-based observational study. BMJ Open 2013;**3**(7). PMID:PMC3731708
33. Kim C, Kang BS, Choi HJ, Lee YJ, Kang GH, Choi WJ, Kwon IH. Nationwide online social networking for cardiovascular care in Korea using Facebook. J Am Med Inform Assoc 2014;**21**(1):17-22. PMID:PMC3912716
