# Supplementary material for: How Health Care Professionals Use Social Media to Create Virtual Communities: An Integrative Review
Source: J Med Internet Res. 2016 Jun 16;18(6):e166. doi: 10.2196/jmir.5312 (PMC4933801; doi:10.2196/jmir.5312)
Supplement: Multimedia Appendix 5 [file jmir_v18i6e166_app5.pdf]

	Quality checklist for questionnaire surveys. [1]
1	Research question and design
1.1	Was there a clear research question, and was this important and sensible?
1.2	Was a questionnaire the most appropriate research design for this question?
2	Sampling
2.1	What was the sampling frame and was it sufficiently large and representative?
2.2	Did all participants in the sample understand what was required of them, and did they attribute the same meaning to the terms in the questionnaire?
3	Instrument
3.1	What claims for reliability and validity have been made, and are these justified?
3.2	*Did the questions cover all relevant aspects of the problem in a non-threatening and on-directive way?*
3.3	Were open-ended (qualitative) and closed-ended questions used appropriately?
3.4	*Was a pilot version administered to participants representative of those in the sampling frame, and the instrument modified accordingly?*
4	Response
4.1	What was the response rate and have non-responders been accounted for?
5	Coding and analysis
5.1	Was the analysis appropriate (eg statistical analysis for quantitative answers, qualitative analysis for open-ended questions) and the correct technique/s used?
5.2	Were outcomes measure by 'blinded' observers or were they objectively verified (eg quantitative measure recorded prospectively and independently)?
6	Presentation of results
6.1	Have all relevant results ('significant 'and 'non-significant')?
6.2	Is there any evidence of data dredging? (ie analyses that were not 'hypothesis driven)?

Author	1.1	1.2	2.1	2.2	3.1	3.2	3.3	3.4	4.1	5.1	5.2	6.1	6.2	quality
Apostolakis 2012 [2]	✓✓✓	✓✓	✓✓✓	✓	✓✓	✓✓	✓	✓✓✓	✓	✓✓	✓	✓✓	No	✓
Cook-Craig 2009 [3]	✓✓✓	✓✓✓	✓✓	✓	✓	✓	✓	×	✓	✓✓	✓	✓✓✓	No	✓
Rodriguez-Recio 2007 [4]	✓✓	✓✓	✓✓✓	×	×	✓✓	✓✓	×	✓	×	✓	✓✓	No	✓
Usher 2012 [5]	✓	✓✓	✓✓	✓	✓✓	✓	✓	×	✓	✓✓	✓	✓✓	No	✓
Watson 2003 [6]	✓	✓	✓✓✓	✓	×	✓	✓	×	✓✓	✓	✓	✓	No	✓
Frisch 2014 [7]	✓	✓	✓	×	×	✓	✓	×	✓	✓	✓	✓	No	✓
Fuoco 2014 [8]	✓✓	✓✓	✓✓	×	×	✓	✓	×	✓✓	✓	✓	✓	No	✓
Ying Mai 2014 [9]	✓	✓	✓	×	×	✓	×	✓	×	✓	×	✓	No	✓
Deen 2013 [10]	✓✓	✓✓	✓✓	✓	✓	✓✓	✓	✓	✓	✓✓	✓	✓	No	✓
Klee 2015 [11]	✓✓	✓	✓✓	✓✓	✓	✓	✓	✓✓✓	✓	✓	✓	✓	No	✓
Tunnecliff 2015 [12]	✓✓	✓✓	✓	✓	✓	✓✓	✓	⚐	⚐	✓	⚐	✓✓	No	✓
Loeb2014 [13]	✓	✓✓	✓✓	✓	✓	⚐	⚐	✓	✓	✓	⚐	✓	No	✓

Author	1.1	1.2	2.1	2.2	3.1	3.2	3.3	3.4	4.1	5.1	5.2	6.1	6.2	quality
Cervantez Thompson 2004 [14]	✓✓	✓✓	✓✓✓	✓	✓✓	✓✓	✓✓	×	✓✓	✓✓	✓	✓✓	No	✓✓
Hoffmann 2011 [15]	✓✓	✓✓	✓✓	✓	×	✓	✓	✓	✓	✓	✓	✓✓	No	✓✓
Kukreja 2011 [16]	✓✓	✓✓	✓	✓	✓✓	✓	✓	✓✓	✓	✓✓	✓	✓✓	No	✓✓
Rolls 2008 [17]	✓✓	✓✓	✓✓✓	×	×	✓✓	✓✓	✓	✓✓	✓✓	✓	✓✓	No	✓✓
Schoch 1997 [18]	✓✓	✓✓	✓✓✓	✓✓	×	×	✓✓	✓✓	✓✓	✓✓	✓	✓	No	✓✓
Shanahan 2009 [19]	✓✓✓	✓✓	✓✓	✓✓	✓	✓	✓	✓✓✓	✓✓	✓✓✓	NA	✓✓	No	✓✓
Widemark 2008 [20]	✓✓	✓✓	✓✓✓	✓✓	✓✓	✓✓✓	✓✓✓	✓✓	✓✓	✓✓✓	✓✓	✓✓	No	✓✓
Kim 2014 [21]	✓✓	✓✓	✓✓	✓✓✓		✓✓	✓✓		✓✓✓	✓✓	✓	✓	No	✓✓
Whitaker 2003[22]	✓✓	✓✓	✓✓	✓	✓✓	✓	✓✓	✓✓	✓✓	✓✓	✓	✓	No	✓✓
Lau 2011 [23]	✓✓✓	✓✓✓	✓✓✓	✓✓	✓	✓✓	✓✓✓	✓✓✓	✓✓✓	✓✓✓	✓✓✓	✓✓✓	No	✓✓✓
McGowan 2012 [24]	✓✓✓	✓✓✓	✓✓	✓✓✓	✓✓✓	✓✓	✓✓	✓	✓✓	✓✓✓	✓✓✓	✓✓✓	No	✓✓✓

References

1. Greenhalgh T, Robert G, Bate P, MacFarlane FKyriakidou O. Diffusion of Innovations in Health Service Organisations: A systematic literature review. Oxford: Blackwell Publishing; 2005. 9780727918697
2. Apostolakis I, Koulierakis G, Berler A, Chryssanthou A, Varlamis I. Use of social media by healthcare professionals in Greece: an exploratory study. International Journal of Electronic Healthcare 2012;**7**(2):105-24. PMID:23079026
3. Cook-Craig PG, Sabah Y. The role of virtual communities of practice in supporting collaborative learning among social workers. British Journal of Social Work 2009;**39**:725-739 DOI:10.1093/bjsw/bcp048.
4. Rodriguez-Recio FJ, Sendra-Portero F. Analysis of the Spanish-speaking mailing list RADIOLOGIA. European Journal of Radiology 2007;**63**:136-143. PMID: 17344009
5. Usher WT. Australian health professionals' social media (Web 2.0) adoption trends: early 21st century health care delivery and practice promotion. Australian Journal of Primary Health 2012;**18**:31-41 PMID:22394660
6. Watson DAR. Ozbug: an email mailing list for physicians that works. Internal Medicine Journal 2003;**33**(11):532-534. PMID:14656258
7. Frisch N, Atherton P, Borycki E, Mickelson G, Cordeiro J, Novak Lauscher H, Black A. Growing a Professional Network to Over 3000 Members in Less Than 4 Years: Evaluation of InspireNet, British Columbia's Virtual Nursing Health Services Research Network. Journal of Medical and Internet Research 2014;**16**(2):e4910. PMID:24566806
8. Fuoco M, Leveridge MJ. Early adopters or laggards? Attitudes toward and use of social media among urologists. BJU International 2015;**115**(3):491-497. PMID:24981237
9. Ying Mai K, Sanghee O. Characteristics of Nurses Who Use Social Media. CIN: Computers, Informatics, Nursing 2014;**32**(2):64-72. PMID:24419089
10. Deen SR, Withers A, Hellerstein DJ. Mental Health Practitioners' Use and Attitudes Regarding the Internet and Social Media. Journal of Psychiatric Practice® 2013;**19**(6):454-463. PMID:24241499
11. Klee D, Covey C, Zhong L. Social media beliefs and usage among family medicine residents and practicing family physicians. Family medicine 2015;**47**(3):222-226. PMID:25853534
12. Tunnecliff J, Ilic D, Morgan P, Keating J, Gaida JE, Clearihan L etal. The Acceptability Among Health Researchers and Clinicians of Social Media to Translate Research Evidence to Clinical Practice: Mixed-Methods Survey and Interview Study. Journal of Medical Internet Research 2015;**17**(5):e119. PMID:4468567
13. Loeb S, Bayne CE, Frey C, Davies BJ, Averch TD, Woo HH, etal. Use of social media in urology: data from the American Urological Association (AUA). BJU international 2014;**113**(6):993-998. PMID:24274744

14. Cervantez Thompson TL, Penprase B. RehabNurse-L: An Analysis of the Rehabilitation Nursing LISTSERV Experience. Rehabilitation Nursing 2004;**29**(2):56-61. PMID:15052747
15. Hoffmann T, Desha L, Verrall K. Evaluating an online occupational therapy community of practice and its role in supporting occupational therapy practice. Australian Journal of Occupational Therapy 2011;**58**:337-345. PMID:21957918
16. Kukreja P, Heck Sheehan A, Riggins J. Use of social media by pharmacy preceptors. Am J Pharm Educ 2011;**75**(9):176. PMID:PMC3230337.
17. Rolls K, Kowal D, Elliott D, Burrell AR. Building a statewide knowledge network for clinicians in intensive care units: Knowledge brokering and the NSW Intensive Care Coordination and Monitoring Unit (ICCMU). Australian Critical Care 2008;**21**(1):29-37. PMID:18226542
18. Schoch NA, Shooshan SE. Communication on a listserv for health information professionals: uses and users of MEDLIB-L. Bulletin of the Medical Library Association 1997;**85**(1):23-32. PMID:PMC226219
19. Shanahan M, Herrington A, Herrington J. The Internet and the medical radiation science practitioner. Radiography 2009;**15**(3):233-241. PMID:25739107
20. Widemark E. Community and Learning: A Virtual Community of Practice for Nurse Practitioners, in Education. 2008, Capella University: Ann Arbour, United States, 8 June 2009. ISBN:9780549573845
21. Kim C, Kang BS, Choi HJ, Lee YJ, Kang GH, Choi WJ, Kwon IH. Nationwide online social networking for cardiovascular care in Korea using Facebook. J Am Med Inform Assoc 2014;**21**(1):17-22. PMID:PMC3912716
22. Whitaker S, Cox AR, Alexander AM. Internet networking for pharmacists: an evaluation of a mailing list for UK pharmacists. International Journal of Pharmacy Practice 2003;**11**(1):25-32. DOI:10.1211/002235702784.
23. Lau ASM. Hospital-Based Nurses' Perceptions of the Adoption of Web 2.0 Tools for Knowledge Sharing, Learning, Social Interaction and the Production of Collective Intelligence. Journal of Medical and Internet Research 2011;**13**(4):e92. PMID:22079851
24. McGowan BS, Wasko M, Vartabedian BS, Miller RS, Freiherr DD, Abdolrasulnia M. Understanding the factors that influence the adoption and meaningful use of social media by physicians to share medical information. Journal of Medical and Internet Research 2012;**14**(5). PMID:23006336
