# Supplementary material for: How Health Care Professionals Use Social Media to Create Virtual Communities: An Integrative Review
Source: J Med Internet Res. 2016 Jun 16;18(6):e166. doi: 10.2196/jmir.5312 (PMC4933801; doi:10.2196/jmir.5312)
Supplement: Multimedia Appendix 6 [file jmir_v18i6e166_app6.pdf]

Multimedia Appendix 6 - Quality assessment of literature reviews [1]

Please note that while studies were assessed against systematic review criteria not all studies claimed to be a one

Reference	Method used	1.1 RQ	1.2 Methodology	1.3 Rigorous search	1.4 Study quality	1.5 Studies are sufficiently similar	score
von Muhlen 2012 [2]	Literature review	poorly addressed	poorly addressed	poorly addressed	adequately addressed	poorly addressed	-
Barnett 2012 [3]	Literature review	well covered	adequately covered	adequately covered	not addressed	well covered	+
Moorhead 2013[4]	Systematic review	well covered	well covered	adequately covered	adequately addressed	adequately covered	++
Hamm 2013 [5]	Scoping review	well covered	adequately covered	well covered	not addressed	well covered	++
Grindrod 2014 [6]	Scoping review	well covered	well covered	adequately covered	not addressed	adequately covered	+
Lawson 2015 [7]	Systematic review	adequately covered	poorly addressed	poorly addressed	not addressed	adequately covered	-
Benetoli 2015 [8]	Systematic review	adequately covered	adequately covered	poorly addressed	not addressed	adequately covered	+
Roberts 2015 [9]	Systematic review	adequately covered	adequately covered	adequately covered	not addressed	adequately covered	+

References

1. SIGN. Scottish Intercollegiate Guidelines Network. no date 8 May 2008]; Available from: http://www.webcitation.org/6cevHavhL http://www.sign.ac.uk/index.html.
2. von Muhlen M, Ohno-Machado L. Reviewing social media use by clinicians. Journal of the American Medical Informatics Association 2012;**19**(5):777-781 PMID:22759618
3. Barnett S, Jones S, Bennett S, Iverson D, Bonney A. General practice training and virtual communities of practice-a review of the literature. BMC family practice 2012;**13**(1):8710. PMID:22905827
4. Moorhead SA, Hazlett DE, Harrison L, Carroll JK, Irwin A, Hoving C. A new dimension of health care: systematic review of the uses, benefits, and limitations of social media for health communication. Journal of Medical and Internet Research 2013;**15**(4):e85. PMID:PMC3636326
5. Hamm MP, Chisholm A, Shulhan J, Milne A, Scott SD, Klassen TP, Hartling L. Social Media Use by Health Care Professionals and Trainees: A Scoping Review. Academic Medicine 2013;**88**(13):1376-1383. PMID:23887004
6. Grindrod K, Forgione A, Tsuyuki RT, Gavura SGiustini D. Pharmacy 2.0: a scoping review of social media use in pharmacy. Research in Social and Administrative Pharmacy 2014;**10**(1):256-270. PMID:23810653
7. Lawson C, Cowling C. Social media: The next frontier for professional development in radiography. Radiography 2015;**21**(2):e74-e80. DOI:http://dx.doi.org/10.1016/j.radi.2014.11.006.
8. Benetoli A, Chen TF, Aslani P. The use of social media in pharmacy practice and education. Research in Social and Administrative Pharmacy 2015;**11**(1):1-46. PMID:24814268
9. Roberts MJ, Perera M, Lawrentschuk N, Romanic D, Papa N, Bolton D. Globalization of Continuing Professional Development by Journal Clubs via Microblogging: A Systematic Review. Journal of medical Internet research 2015;**17**(4)10. PMID:4424319
