# Supplementary material for: How Health Care Professionals Use Social Media to Create Virtual Communities: An Integrative Review
Source: J Med Internet Res. 2016 Jun 16;18(6):e166. doi: 10.2196/jmir.5312 (PMC4933801; doi:10.2196/jmir.5312)
Supplement: Multimedia Appendix 7 [file jmir_v18i6e166_app7.pdf]

Multimedia appendix 7 - Content of posts on healthcare social media

Study	Manifest Content	Latent Content	Posting behaviour
Brooks<br>Midwives<br>United Kingdom<br>Public Health organisation [1]		Knowledge work Φ – 88% (172)<br>- Tacit knowledge – 20% (39)<br>- Explanatory force to suggested resolution 20% (39)<br>Information work Ψ – 9% (16)<br>Dispersed leadership and collegial support – 32.6% (63)	44 of 96 staff<br>- 18 staff midwives posted 51% (36/70)<br>- 6 graded ≥ sister<br>- 3 community midwives<br>- 17 posted without demonstration
Brooks<br>Cardiology and geriatric Nurses; Midwives<br>United Kingdom<br>Public Health organisation [2]	Cardiology - 15 months<br>- 21 threads with 71 posts<br>Older persons – 7.5 months<br>- 6 threads with 18 posts<br>Obstetrics – 1.5 months<br>- 11 threads with 70 posts	Cardiology<br>- Knowledge work Φ – 30%<br>- Information work Ψ -70%<br>Older persons<br>- Knowledge work Φ – 74%<br>- Information work Ψ – 26%<br>Obstetrics<br>- Knowledge work Φ – 92%<br>- Information work Ψ – 8%	Cardiology - 26 contributors<br>- 15 RN (<2yrs); 8 RN- E; 9 experienced RNs; 1 nurse manager<br>Older persons<br>– 11 contributors<br>- 1 RN <2yrs experience; 10 experienced RNs; 1 nurse manager<br>Obstetrics – 29 contributors<br>- 18 midwifes; 3 community midwives;2 incharge midwives; 4 midwifery managers
Berman<br>Social workers<br>Israel<br>Two topic specific Lists [3]	Email traffic<br>- List 1 – 369 emails/37 days<br>- List 2 - 194 emails/43 days	Discussion of issues:<br>- List 1 43.4%; List 2 56.3%<br>Information request only:<br>- List 1 22.8%; List 2 10.9%<br>Information transfer only:<br>- List 1 10.7%; List 2 12.5%	List 1 168 posters<br>- 38 posters/ 58.8% of emails<br>List 2 – 64 posters<br>- 8 participants / 51% of emails
Bowers<br>Psychiatric nursing<br>United Kingdom – International [4]	Email traffic – 16 months<br>- Threads <3 emails = 130<br>- Threads > 3 emails = 45 (range 4-33)	Topics of importance:<br>- Nursing models – 33 emails/ Advanced Practice – 27/ Lurking – 26/ Mental health laws – 23/ Labels (of patients) – 22/ Violence - 22	Not described
Thomas<br>General practitioners<br>United Kingdom [5]	May 1995 Traffic<br>- 155 emails (daily average 5)<br>January 1998<br>- 1160 (daily average 40)	13 Topics – humor 283, technical 232, clinical 226<br>- From survey Topics of greatest value: General discussion (75%); camaraderie (17%); clinical discussion (3%)	May 1995<br>- 59 posters; lurking 62%<br>January 1998<br>- 182 posters; lurking 71%<br>Range – 1 email/65 ;2-10 emails/392; 11-30 emails/12; >30/4, one person sent 14
Reutzel<br>School nurses<br>US-International<br>Professional organisation US [6]	Email traffic – 5 months<br>71 threads on medication management<br>Emails per thread 1-27	Components of medication administration that present as problems<br>- Administration 21%; Therapeutic appropriateness 19%; Use 17%; Storage 13%; Documentation 9%; Transfer 7.5%;	Not evaluated

Study	Manifest Content	Latent Content	Posting behaviour
		Liability 7%; Information 2%<br>- Sources of Authority<br>- Practices 37 %; Opinions 28%; Policies 17%; Legalities 15%; guidelines 3%	
Cervantez<br>Rehabilitation nurses<br>US-International<br>Professional organisation US<br>[7]	Email traffic – 18 months<br>- 2053 emails<br>- 551 initiations<br>- 1678 responses<br>- 178 both	- Administration -29%<br>- Clinical procedures – 26%<br>- Professional issues – 16%<br>- Impairment/disability – 15%<br>- Clinical documentation – 8%<br>- Miscellaneous – 5%	- 67% (318/475) posted Range 1-91 (mean 6.4)<br>- 27.8% - 1 post<br>- 10 members - > 30 posts<br>- 551 initiated postings
Smith<br>Medical librarians<br>United States [8]	Email traffic<br>- 1991 – 123 ; 1992 – 162; 2002 – 201<br>Singleton messages<br>- 1991 – 22%; 1992 - 42.7%; 2002 – 68.2%<br>- Thread length – mean(SD)<br>- 1991 – 4.2 (1.6); 1992 – 2.8 (1);2002 -2.1 (0.2)	- Discussion (exchange of opinion, practices, product evaluation)<br>○ 1991 – 58.5%; 1992 -35.2%; 2002 – 21.4%<br>- Information exchange<br>○ 1991 – 13.5%; 1992 -22.2%; 2002 – 49.3%<br>- Meta discussion (comments on List itself)<br>○ 1991 – 8.1%; 1992 -6.8%; 2002 – 0%<br>- Noise<br>○ 1991 – 8.9%; 1992 -11.7%; 2002 – 2%<br>- Thanks<br>○ 1991 – 0.8%; 1992 -1.9%; 2002 –16.9%<br>- Miscellaneous<br>○ 1991 – 10.6% ; 1992 -22.2%; 2002 – 10.5%	- Member contributions<br>- 1991 – 6% (n=5) → 30.1%<br>- 1992 – 12.1% (n=13) → 29.6%<br>- 2002 – 15.5% (n=20) → 29.4%
[9]<br>Advanced practice critical care nursing<br>United States	Email traffic – first two weeks of each month for 12 months - 1059 emails<br>Query to response rate: 1.68-1	Types of online activities - 1119 thematic units<br>- Sharing knowledge - 56.2%; Solicitation – 33.4 %; Job posting – 6.1 %; miscellaneous 3.3%<br>Types of knowledge shared<br>- Book knowledge – 8.7%<br>- Practical knowledge – 92.3% : Institutional practice – 53.5%; Personal opinion – 24.7%; Personal suggestion – 13.2; Cultural knowledge - 0	Not measured
Hew<br>Advanced practice critical care nursing<br>United States [10]	Email traffic – 6 weeks (first two weeks of March 2003-2006)	Types of knowledge shared<br>- Institutional practice 57.3%<br>- Personal opinion - 26.3%<br>- Personal suggestion 10.3%<br>- Book knowledge 6.1%	Not measured

Study	Manifest Content	Latent Content	Posting behaviour
Rodriguez-Recio<br>Radiology professionals<br>Spain<br>Professional organisation [11]	1998-2003 – 2700 emails<br>Distribution<br>- Monthly mean 44.3 (SD 26, range 0-107)	Categories<br>- Scientific information 43.4% (n=1185) 130 subjects identified<br>- Information request 24.7% (n=293)<br>- Answer – 53.8% (n=638)<br>- Information spreading 21.4 (n=254)<br>- Discussion – 21.7 (n=592)<br>- Announcement – 15.7% (n= 436)<br>- Noise – 13.5% (n=368)<br>- Clinical case – 5.4% (n=148)	No of messages - % of subscribers (n=)<br>- >200 - 0.3 (1)<br>- 41-50 - 1.3 (5)<br>- 31-40 - 1.6 (6)<br>- 21-30 - 2.4 (9)<br>- 11-20 - 5.5 (21)<br>- 5-10 - 8.5 (32)<br>- 2-4 - 20.6 (78)<br>- 1 only - 13.5 (51)<br>- None - 46.5 (175)
Macdonald<br>Travel medicine<br>Multi-disciplinary<br>International<br>Professional organisation [12]	Email traffic<br>- 1710 messages over 8 months<br>- Primary – 26% (n=389)<br>- Response – 74% (n=1120)<br>- Mode – 1 (Range 1-51)	Message types<br>- Administration – 12% (n=204)<br>- Educational – 88% (n=1506)<br>Topics - 27<br>- Vaccine preventable diseases – 37%<br>- Vector-borne diseases – 22%<br>- General – 16%<br>- Pre-travel – 12%<br>- Miscellaneous - 13%	369 users (41% of List members)<br>- Doctors - 68% (n=252) – 68% posts<br>- Nurses – 22% (n=80) – 27% posts<br>- Location: US & Canada – 65%; Europe – 18%; Oceania – 6%; Asia – 5%; Africa – 2%<br>Users<br>- 20 most frequent users – 43% total posts<br>- 10 most frequent users – 30% total posts
Morken<br>Occupational health (OH)<br>Multidisciplinary<br>Norway<br>Professional organisation [13]	Email traffic<br>- 1997-2006 -5269 emails –<br>- Messages per year:1997-417; 2000-746; 2006 -315	- Chemical hazards – 19% (n=1001)<br>- Organisation of OH services – 17% (n= 890)<br>- Methods in health, safety and environment – 10% (n=554)<br>- Ergonomics – 8% (n=436)<br>- Noise and radiation – 5% (n=5)<br>August-October 2006 activity<br>- 46 subjects: 28 posed as questions; 13 as pure information; 5 irrelevant<br>- Of questions: 64% (n=18) were answered satisfactorily; 32% (n=9) partially answered; 18% (n=5) were not answered	Messages per list member: 1997-2.1; 2004-0.6<br>- Contributors (n=132): occupational hygienists (27%); doctors (14%); physiotherapists (13%); nurses (13%)<br>- Mean messages per contributor: 2.4 (range 1-20)<br>- 2% (11/467 list members) contributed 26% (n=83) messages
Long<br>Paediatric occupational therapists<br>Australia<br>Healthcare organisation [14]	Email traffic – 12 months<br>- 2104 posts [843 initial posts)<br>- Mean reciprocity – 2.5 (SD 2.8)	- Practice and organisational – 26.69% (n=225)<br>- Performance component– 21.12% (n=178)<br>- Performance area – 17.91% (n=15)<br>- Health conditions – 6.76%(n=57)	430 of possible 600 posted<br>- Low level (< 4) – n=239 contributed 20.48% of total posts<br>- Medium level (4-20) – n=179 contributed 62.32% of total posts<br>- High level (19-59) – n=12 contributed 17.2% of total posts
Cook-Craig	- Jan 07 - 10 discussion forums with 1750 hits	Not evaluated	Jan 07 - 200 users/1000 members

Study	Manifest Content	Latent Content	Posting behaviour
social workers<br>Israel<br>Public Health organisation<br>[15]	- Jan 08 – across 16 discussion forums with 6800 hits<br>- May 08 – across 18 discussion forums with 7000		- 8.75 hits/user<br>Jan 08 - 1200 users/3000 members<br>- 5.66 hits/user<br>May 08 – 1500 users/4200 members<br>- 4.66 hits/user
Franko<br>Twitter<br>Orthopedic [16]	-	- Last 10 tweets - 64<br>- News - 71.8%<br>- Personal - 56.2%<br>- Professional - 37.5<br>- Opinions - 12.5%<br>- Product/promotion - 6.3<br>- Other - 9.4	- 1 tweet % n=69<br>- Within 1 week - 34.7%<br>- Within 1 month - 46.3<br>Within last year - 71%
Foong<br>India<br>Plastic surgeons<br>Discussion forum [17]	2217 emails in a calendar year<br>330 discussion threads (average 6.7 emails per)	Categories - 4<br>1. Advice on treatment – 40%<br>2. Education (meetings; courses; fellowships) – 25%<br>3. Case reports – 25%<br>4. Introduction of new members – 4%<br>Topics<br>1. Training & courses – 26.7%<br>2. Cleft – 15.4%<br>3. Aesthetics – 13.1%<br>4. Trauma – 12.5%<br>5. Head & Neck – 8.4%<br>6. Cutaneous – 6.4%<br>7. Perineal/genital – 6.1%<br>8. Scar – 4.7%<br>9. Other – 6.7%	Not evaluated
Abrahamson<br>International discussion forum for nurses<br>Themed forums [18]	294 discussion threads – over one month<br>-	25 categories<br>1. Technical or Clinical – 17%<br>2. Non-clinical – 83% - 24 categories<br>a. Career advice – 26% (75)<br>b. Education advice – 8% (24)<br>c. Perception of unjust treatment by management – 7% (21)<br>d. Shift work – 5% (14)<br>e. Handling job related emotions – 4% (13)<br>f. Sharing stories – 4% (13)<br>g. Nursing management – 4%(12)other	Not evaluated

Study	Manifest Content	Latent Content	Posting behaviour
Dieleman<br>United kingdom<br>Forensic occupational therapist [19]	2494 posts over 8 years (monthly median 303 IQR 227-424)	Themes:<br>1. Seeking & giving advice - 40.5% (n=1010)<br>2. Requesting and sharing material resources - 19.4% (n=485)<br>3. Networking - 27.3% (n=680)<br>4. Defining the OT role – 8% (n=199)<br>5. Student posts - 5.2% (n=129)	Post origin Location<br>▪ UK – 60% (n = 1485)<br>▪ Unknown – 32% (n=810)<br>▪ 20 other countries – 8% (n=199)
[20]<br>Paediatric pain forum<br>Multidisciplinary	568 posts over 115 threads<br>Average thread length 4.94 (range 1-25)<br>•		46 unique members<br>31 posters<br>Posters<br>• 12 ≥ 10 posts<br>• Nurses - 77% posts<br>Reading<br>• 26 < 25 reads<br>• 8 – 25-44 reads<br>• 12 – 45-94 reads<br>• Nurses - 67%
Whitaker<br>Pharmacy<br>Listserv<br>United Kingdom [21]	One month 386 covering 429 topics	Topics<br>1. Patient problems/clinical problems 20% (n=87)<br>2. Pharmacy politics 18% (n=77)<br>3. Non-pharmacy chat 14% (n=60)<br>4. Legal issues 9% (n=37)<br>5. Drug Tariff 7% (n=30)<br>6. Government policy 5% (n=21)<br>7. Business/finance 5% (n=22)<br>8. Risk management 4% (n=17)<br>9. Supportive 3% (n=14)<br>10. Non-pharmacy It 2% (n=7)<br>11. Pharmacy IT 1% (n=4)<br>12. Other 12% (n=53)	
Desai 2012<br>North America<br>Multi-disciplinary nephrology Twitter [22]	993 tweets<br>Informative 29%<br>Uninformative 38%	Sentiment score<br>Mean 0.094 (SD 0.476; range -1.70-2.67)	993 tweets by 172 (1.4% of participants)
Hajar 2014		Exclusively social 57% (n=115	Daily tweets mean (SD) – 3 ± 7

Study	Manifest Content	Latent Content	Posting behaviour
Twitter<br>USA[23]		Predominantly social 33% (n=68)<br>Predominantly professional 9% (n=18)<br>Exclusively professional 1% (n=3)	Reach mean (SD) – 399 ± 1163
Kim 2014<br>Korea<br>Facebook<br>Emergency physicians [24]	Cat 1 – Asking for clinical advice on a difficult case to aid decision making – 26.7% (74/277) – 667 comments; median 6(IQR 3-10); difficult ECGs 54% and acute coronary syndrome 32.4%)<br>Cat 2 posts discussing interesting cases 50.5% (140/277)<br>Cat 3 Educational (informative knowledge, sharing references or informative websites - 15.9 % (44/277)<br>Cat 4 - announcements - 6.9% (19/277)		277 index posts<br>Posts per member not quantified<br>Identified 14 major respondents however did not quantify
Moorley 2014<br>Twitter<br>Nurses [25]			Tweet chats<br>Dignity – 75 participants<br>The new vision for nursing – the vision and 6Cs – 89 participants (1700 website pages visits)<br>The new vision for nursing – obstacles and solutions – 72 participants (1610 website pages visits)<br>Inpatient use of social media – 140 participants (2930 website pages visits)<br>Defining sage staff levels – 95 participants (1403 website pages visits)<br>The Francis report – 93 participants (1322website pages visits)
Ferguson 2014<br>Australia and New Zealand<br>Twitter<br>Cardiac professionals [26]			Origin of tweets<br>Australia - 68.9% (514/779)<br>USA – 28.4% (212/779)<br>UK – 1.6% (12/779)<br>5 other countries – (8/779)<br>Top ten tweets (range) – 14-83<br>Top ten username mentioned – (range) 30-151<br>Top ten impressions (range) – 9034 - 979 100
Mishori 2014<br>USA<br>4 medical professional			High dissemination potential (AMA 122 066 397; AAP 14 496 559; ACP 11 228 160; AAFP – 6 959 092)

Study	Manifest Content	Latent Content	Posting behaviour
societies<br>One general (AMA), three specialialty (AAFP, AAP and ACP)			Low actual dissemination
Anderson 2014<br>Australia<br>Public health<br>Twitter [27]	748 tweets<br>13 broad themes<br>1. Statement from keynote & plenary session - 37.3% (282)<br>2. Concurrent sessions – 14.2% (107)<br>3. What is happening next and looking forward to – 12.2% (92)<br>4. Personal comments, likes and dislikes – 8.2% (62)<br>5. Pre-conference workshop (aboriginal and Torres strait islander – 6.7% (51)<br>6. AHPA and Croakey - 6.6% (50)<br>7. Workshops - 4% (30)<br>8. General appreciative remarks – 3.6% (27)<br>9. Health information not from conference – 2.5% (19)<br>10. Statistical facts - 2.2 (17)<br>11. Conference oration - 1.3% (10)<br>12. Not categorized – 0.7 (5)<br>13. Posters – 0.5 (4)		12.8% of attendees (96 tweeters/466? = 20%)<br>Range 1-129<br>75% < 6<br>9% >20 (129, 82, 50, 36
Mishori 2014<br>USA<br>Physicians<br>Twitter[28]	1818 by 181 user accounts<br>Tweet types<br>• Original – 61% (1103)<br>• Modified – 9% (159)<br>• Re-Tweet – 31% (556)<br>Original tweet content<br>• Session related 69.7%<br>• Social – 14.2%<br>• Logistics – 6.9%<br>• Other – 7.6%<br>Advertisement - 1.6%		13% of attendees (181 /1370)<br>Top tweeter – 15.4% of all tweets<br>Top 10<br>– 56% of data<br>– Range – 61-280
Hawkins 2014<br>International<br>Radiology professionals<br>Twitter [29]	Tweets – 2011 4061; 2012 5630<br>Characteristics<br>• Meeting related with links – 201150% (n=1630); 2012 55% (n=2443<br>• Original tweets – 2011 62% (n=2015) ; 2012		N tweeting – 2011_755; 2012-1116<br>Tweets per participant: mean 4.9 both years<br>• 2011 range 1-156; SD 12.4;<br>• 2012 range 1-235; SD 15.1<br>• > 5 tweets 2011-135; 2012-179

Study	Manifest Content	Latent Content	Posting behaviour
	63% (n=2816)		• Top 100 2011 mean 22(range 6-156); 2012 mean 29 (range 8-235) ; + 31.8%<br>User origin<br>• 2011: USA 67%; Europe 17%; Asia 5%; South America 3%; Australia 0.6%; Africa 0.4%<br>• 2012: USA 65%; Europe 18%; Asia 5%; South America 2%; Australia 2%; Africa 0.4%
Matta 2014<br>North America<br>Physicians<br>Twitter[30]	Tweets<br>#uro12 n=756; (Retweets 17.8% , Informative 21.8% , uninformative 60.4%)<br>#aua13 – 3956 (Retweets 24.9%, Informative 29.4%, uninformative 45.3%)<br>#cua2012 – 635 (Retweets 19%, Informative 44.8% , uninformative 36.2%)<br>#cua2013 – 5402 (Retweets 27.2%, Informative 38.6% , uninformative 34.2%		N tweeting<br>• 2012-134<br>• 2013-540
Canvasser 2015<br>North America<br>Physicians<br>Twitter [31]	335 tweets (217 primary; 118 retweets)<br>Informative 56.7% (n=190; 39.5%)<br>Uninformative 44.3%	Tweet sentiment – mean 0.13 (range -0.90-1.80); 57% neutral	N tweeting – 68 (42 at meeting); 4.9 per (range 1-55)<br>Tweets for top ten tweeters<br>• Range 2-55<br>• Responsible for 64% tweets (87% primary; 35% retweets<br>Tweeting conference faculty 3.3% (10/302)<br>Tweet reach 38 141
Context: healthcare professional type; country; initiating organisation;	Φ Knowledge work- posts with critical reflection<br>Ψ Information work – posts without critical reflection	Personal opinion – individual opinion (I believe …)<br>Person suggestion – personal solution for problem or issue (whenever I teach a topic I…)<br>Institutional practice – knowledge related to an institution (our hospital ..)<br>Book knowledge individual's awareness of knowledge about mere facts such as statutes, policies, and standards.	AMA – American medical association<br>ACP – American college of physicians<br>AAP – American Academy of Pediatrics<br>AAFP – American Academy of Family Practice

References

1. Brooks F, Scott P. Exploring knowledge work and leadership in online midwifery communication. Journal of Advanced Nursing 2006;**55**(4):510-20 PMID:16866846
2. Brooks F, Scott P. Knowledge work in nursing and midwifery: an evaluation through computer-mediated communication. International Journal of Nursing Studies 2006;**43**:83-97 PMID:16326164
3. Berman Y. Discussion groups on the Internet as sources of information: the case of social work. Aslib Proceedings 1996;**48**(2):31-36. http://dx.doi.org/10.1108/eb051407.
4. Bowers L. Constructing international professional identity: what psychiatric nurses talk about on the Internet. International Journal of Nursing Studies 1997;**34**(3):208-212. PMID: 9219053
5. Thomas RE, James SD. Informal communications networking health professionals: a study of gp-uk. Health Informatics Journal 1999;**5**(2):74-81. DOI:10.1177/146045829900500204.
6. Reutzel TJ, Patel R. Medication management problems reported by subscribers to a school nurse listserv. The Journal of School Nursing 2001;**17**(3):131-139. PMID:11885443
7. Cervantez Thompson TL. You've got mail: Rehabilitation nurses on the RehabNurse-L LISTSERV. Rehabilitation Nursing 2002;**27**(4):146-151. PMID:12116527
8. Smith C. A longitudinal study of the culture of MEDLIB-L. Journal of Hospital Librarianship 2004;**4**(1):29-42. PMID:14656258
9. Hara NHew K. Knowledge-sharing in an online community of health care professionals. Information, Technology and People 2007;**20**(3):235-261 DOI:10.1108/09593840710822859.
10. Hew KF, Hara N. An online listserv for nurse practitioners: a viable venue for continuous nursing professional development? Nurse Education Today 2008;**28**(4):450-71 PMID:17881096
11. Rodriguez-Recio FJ, Sendra-Portero F. Analysis of the Spanish-speaking mailing list RADIOLOGIA. European Journal of Radiology 2007;**63**:136-143 PMID: 17344009
12. Macdonald L, MacPherson DW, Gushulak BD. Online communication as a potential travel medicine research tool: analysis of messages posted on the TravelMed listserv. Journal of Travel Medicine 2009;**16**(1):7-12. PMID:19192121
13. Morken T, Bull N, Moen BE. The activity on a Norwegian Occupational Health mailing list 1997-2006. Occupational Medicine 2009;**59**:56-58. PMID:19001070
14. Long S, de Jonge D, Ziviani JJones A. Paediatricots: utilisation of an Australian list serve to support occupational therapists working with children. Australian Occupational Therapy Journal 2009;**56**(1):63-71 PMID:20854490
15. Cook-Craig PG, Sabah Y. The role of virtual communities of practice in supporting collaborative learning among social workers. British Journal of Social Work 2009;**39**:725-739. DOI:10.1093/bjsw/bcp048.
16. Franko OI. Twitter as a communication tool for orthopedic surgery. Orthopedics 2011;**34**(11):873-876. PMID:22050252
17. Foong DP, McGrouther DA. An Internet-based discussion forum as a useful resource for the discussion of clinical cases and an educational tool. Indian journal of plastic surgery, 2010;**43**(2):195. PMID:PMC3010782
18. Abrahamson K, Fox R, Anderson JG. What nurses are talking about: content and community within a nursing online forum. Stud Health Technol Inform 2013;**183**:350-5. DOI:10.3233/978-1-61499-203-5-350.
19. Dieleman C, Duncan EA. Investigating the purpose of an online discussion group for health professionals: a case example from forensic occupational therapy. BMC health services research 2013;**13**(1):253. PMID:PMC3702402
20. Stewart SA, Abidi SSR. Applying social network analysis to understand the knowledge sharing behaviour of practitioners in a clinical online discussion forum. Journal of medical Internet research 2012;**14**(6):e170. PMID:PMC3799555
21. Whitaker S, Cox AR, Alexander AM. Internet networking for pharmacists: an evaluation of a mailing list for UK pharmacists. International Journal of Pharmacy Practice 2003;**11**(1):25-32. DOI:10.1211/002235702784.
22. Desai T, Shariff A, Shariff A, Kats M, Fang X, Christiano C, Ferris M. Tweeting the meeting: an in-depth analysis of Twitter activity at Kidney Week 2011. PloS one 2012;**7**(7):e40253. PMID:PMC3390326
23. Hajar Z, Clauson KA, Jacobs RJ. Analysis of pharmacists' use of Twitter. American Journal of Health-System Pharmacy 2014;**71**(8):615-619. PMID:24688034
24. Kim C, Kang BS, Choi HJ, Lee YJ, Kang GH, Choi WJ, Kwon IH. Nationwide online social networking for cardiovascular care in Korea using Facebook. J Am Med Inform Assoc 2014;**21**(1):17-22. PMID:PMC3912716

25. Moorley CR, Chinn T. Nursing and Twitter: Creating an online community using hashtags. Collegian 2014;**21**(2):103-109. PMID:25109208
26. Ferguson C, Inglis SC, Newton PJ, Cripps PJS, Macdonald PS, Davidson PM. Social media: A tool to spread information: A case study analysis of Twitter conversation at the Cardiac Society of Australia and New Zealand 61st Annual Scientific Meeting 2013. Collegian 2014;**21**(2):89-93. PMID:25109206
27. Anderson G, Gleeson S, Rissel C, Wen LMBedford K. Twitter tweets and twaddle: twittering at AHPA. Health Promotion Journal of Australia 2014;**25**(2):143-146. PMID:25200470
28. Mishori R, Levy BDonvan B. Twitter Use at a Family Medicine Conference: Analyzing# STFM13. Family medicine 2014;**46**(8):608-614. PMID:25163039
29. Hawkins CM, Duszak RRawson JV. Social Media in Radiology: Early Trends in Twitter Microblogging at Radiology's Largest International Meeting. Journal of the American College of Radiology 2014;**11**(4):387-390. PMID:24139963
30. Matta R, Doiron C, Leveridge MJ. The Dramatic Increase in Social Media in Urology. The Journal of Urology 2014;**192**(2):494-498. PMID:24576656
31. Canvasser NE, Ramo C, Morgan TM, Zheng K, Hollenbeck BK, Ghani KR. The use of social media in endourology: an analysis of the 2013 World Congress of Endourology meeting. Journal of Endourology 2015;**29**(5):615-620. PMID:25026076
